# Classical theories of gravity produce entanglement

**DOI:** 10.1038/s41586-025-09595-7

**Published:** 2025-10-22

**Authors:** Joseph Aziz, Richard Howl

**Affiliations:** Department of Physics, Royal Holloway, University of London, Egham, UK

**Keywords:** Quantum information, General relativity and gravity

## Abstract

The unification of gravity and quantum mechanics remains one of the most profound open questions in science. With recent advances in quantum technology, an experimental idea first proposed by Richard Feynman^1^ is now regarded as a promising route to testing this unification for the first time. The experiment involves placing a massive object in a quantum superposition of two locations and letting it gravitationally interact with another mass. If the two objects subsequently become entangled, this is considered unambiguous evidence that gravity obeys the laws of quantum mechanics. This conclusion derives from theorems that treat a classical gravitational interaction as a local interaction capable of transmitting only classical, not quantum, information^2–8^. Here we extend the description of matter used in these theorems to the full framework of quantum field theory, finding that theories with classical gravity can then transmit quantum information and, thus, generate entanglement through physical, local processes. The effect scales differently to that predicted by theories of quantum gravity, and so it gives information on the parameters and form of the experiment required to robustly provide evidence for the quantum nature of gravity.

## Abstract (web summary)

By applying the full framework of quantum field theory, it is shown that local classical theories of gravity can transmit quantum information and, thus, generate entanglement through physical, local processes.

## Main

Although the other fundamental interactions—electromagnetism and the strong and weak forces—have been successfully married to quantum theory, the standard methods of quantization seem to fail for gravity^9^. This has motivated alternative approaches to the unification of gravity with quantum theory, including string theory, loop quantum gravity and proposals that gravity is not quantized at all but remains fundamentally classical^10^. A decisive factor in determining which route is correct has so far been lacking: experimental evidence. At the 1957 Chapel Hill conference, Feynman proposed a thought experiment that could reveal the quantum nature of gravity^1^, an idea now becoming feasible through rapid progress in quantum experiments^5,6^. In Feynman’s proposal, an object of Planck mass (0.02 mg) is placed in a quantum superposition of two locations before interacting gravitationally with another mass^1^. Although Feynman’s exact measurement prescription for then determining the quantum nature of gravity is unclear from the original conference transcript^1^, today this is considered as the observation of entanglement between the massive objects, with several theorems on how physically realistic (local) classical theories of gravity can never create entanglement between the massive objects^2–8^. These theorems rest on the assumption that theories of classical gravity can involve only local operations and exchanges of classical information^2–8^. This is because non-local, action-at-a-distance processes are considered unphysical, and it seems natural that a classical gravitational interaction cannot transmit quantum information. Under this assumption, the interaction falls into a class of processes that, according to quantum information theory or generalizations^7,8^, cannot create entanglement, formalized as local operations and classical communication (LOCC) in quantum information theory^3,5^.

A substantial number of experimental proposals have been developed for witnessing this gravitationally induced entanglement^11–13^, and initial work on such experiments is underway^11,14–17^. Owing to the fundamental significance of the experiments, several works have appeared on whether entanglement can really provide evidence for quantum gravity, which are often inspired by discussions at the Chapel Hill conference^1^. These works have focused on whether classical gravity can act through non-local operations violating the LO part of LOCC and, thus, whether classical gravity can generate entanglement through non-local means (for a review, see refs. ^12,18^ and Supplementary Information). However, this goes against our understanding of interactions in nature acting locally, whether they are based on electromagnetism, the Standard Model or general relativity, and is, thus, generally ruled out on physical grounds^2,3,5,6,18,19^.

Assuming the LO part of LOCC on physical grounds, leaves the CC part. The idea that a classical theory of gravity should involve only classical communication seems natural and has not been questioned. However, here we show that local classical theories of gravity can, in fact, generate quantum communication and, thus, entanglement. The arguments and theorems for classical gravity operating only as LOCC implicitly treat matter in standard quantum mechanics. However, to the best of our knowledge, matter obeys quantum field theory (QFT), and when this is taken into account, we show that a classical gravity interaction naturally gives rise to quantum communication.

## Quantum electrodynamics

**Fig. 1:**
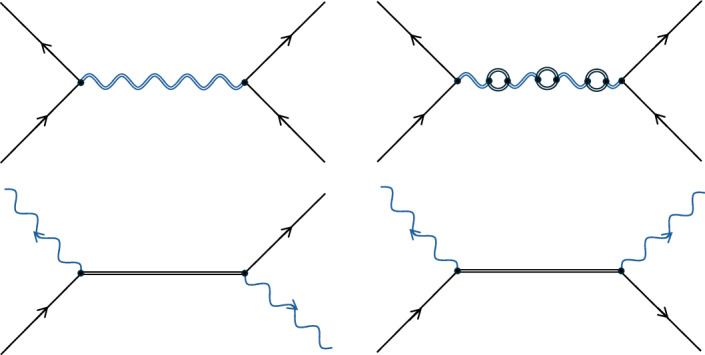
Feynman diagrams for QED or linear quantum gravity. Wiggly blue lines represent photons or gravitons. Black lines represent electrons, positrons, or general matter or antimatter particles. For ease of visualization, double lines without arrows represent virtual particles.

To better understand how classical gravity theories can generate quantum communication, we first review quantum electrodynamics (QED). This is our best theory for how matter interacts through electromagnetism, and it involves treating both matter and the electromagnetic field with QFT. The interaction Hamiltonian is

1 $${\widehat{H}}_{{\rm{int}}}^{{\rm{QED}}}=\int {{\rm{d}}}^{3}{\bf{x}}\,q{\widehat{A}}_{\mu }({\bf{x}})\widehat{\overline{\psi }}({\bf{x}}){\gamma }^{\mu }\widehat{\psi }({\bf{x}}),$$

where $\widehat{\psi }({\bf{x}})$ is a charged fermionic field with *q* its charge, $\widehat{\overline{\psi }}({\bf{x}})$ is the Dirac adjoint field, ${\widehat{A}}_{\mu }({\bf{x}})$ is the quantized 4-potential and *γ*^*μ*^ are the gamma matrices. Calculations with QED are mostly performed perturbatively, where we can intuitively view interactions through Feynman diagrams. For example, Fig. 1 (top left) describes an interaction between two electrons at first order in perturbation theory. In this diagram, the electromagnetic interaction between the electrons is mediated by a virtual photon, which can be viewed as quantum communication^2,20^.

However, a critical finding of QED compared with classical electromagnetism is that the interaction processes need not be mediated by just virtual photons. For example, at higher order in the electron-scattering process, there are diagrams such as Fig. 1 (top right) in which the virtual photon is accompanied by virtual electron particles. In fact, even at leading order there are processes that involve only virtual electron propagators, some of which are depicted in Fig. 1 (bottom row). When viewed from a non-perturbative perspective, there is no way to separate virtual matter from virtual photons in QED, and all electromagnetic interactions should be viewed as a combination of matter and electromagnetic fields propagating the interaction^21^.

## Perturbative quantum gravity

Although there is no consensus on a full theory of quantum gravity, at low energies it is widely accepted that such a theory should approximate perturbative quantum gravity^13,22–25^. This is an effective field theory that involves the quantization of linear general relativity with matter fields: the full space-time metric *g*_*μν*_ is broken up into *η*_*μν*_ + *h*_*μν*_, with *η*_*μν*_ the metric of a background classical space-time and ∣*h*_*μν*_∣ ≪ 1, which is quantized. The interaction Hamiltonian for matter interacting with gravity is

2 $${\widehat{H}}_{{\rm{int}}}^{{\rm{QG}}}=-\,\frac{1}{2}\int {{\rm{d}}}^{3}{\bf{x}}\,{\widehat{h}}^{\mu \nu }({\bf{x}}){\widehat{T}}_{\mu \nu }({\bf{x}}),$$

where ${\widehat{T}}_{\mu \nu }({\bf{x}})$ is the quantized energy–momentum tensor for matter. For example, describing matter with a massive complex scalar field $\widehat{\phi }$, ${\widehat{T}}_{\mu \nu }({\bf{x}})$ becomes

3 $$\begin{array}{l}{\widehat{T}}_{\mu \nu }({\bf{x}})={\widehat{{\mathcal{T}}}}_{\mu \nu }[{\widehat{\phi }}^{\dagger }({\bf{x}})\widehat{\phi }({\bf{x}})]\\ \,\,:= \,{\partial }_{\{\mu }{\widehat{\phi }}^{\dagger }{\partial }_{\nu \}}\widehat{\phi }-{\eta }_{\mu \nu }{\partial }_{\rho }{\widehat{\phi }}^{\dagger }{\partial }^{\rho }\widehat{\phi }-{\eta }_{\mu \nu }\frac{{m}^{2}{c}^{2}}{{\hbar }^{2}}{\widehat{\phi }}^{\dagger }\widehat{\phi },\end{array}$$

where *m* is mass, *c* the speed of light and *ħ* the Planck constant. Here, ${\partial }_{\{\mu }A\,{\partial }_{\nu \}}B:= {\partial }_{\mu }A\,{\partial }_{\nu }B+{\partial }_{\nu }A\,{\partial }_{\mu }B$. The interaction Hamiltonian in equation (2) is of similar form to that in equation (1), and perturbative quantum gravity parallels QED closely^18^, with photons essentially being replaced with gravitons. For example, for two matter particles interacting, then at first order we have a diagram such as Fig. 1 (top left) but with a virtual graviton mediating the interaction rather than a virtual photon. Similarly, we also have all the other diagrams in Fig. 1, where there are virtual matter or virtual graviton propagators.

## Perturbative classical gravity

In a fundamentally classical theory of gravity, the gravitational field is classical. Therefore, in the low-energy regime, most simply we would have an interaction Hamiltonian as equation (2) but with *h*_*μν*_ not quantized:

4 $${\widehat{H}}_{{\rm{int}}}^{{\rm{CG}}}=-\,\frac{1}{2}\int {{\rm{d}}}^{3}{\bf{x}}\,{h}^{\mu \nu }(x)\,{\widehat{T}}_{\mu \nu }({\bf{x}}).$$

This is the interaction Hamiltonian of QFT in linear curved space-time, which, like any other QFT, is a local theory^26–28^.

**Fig. 2:**
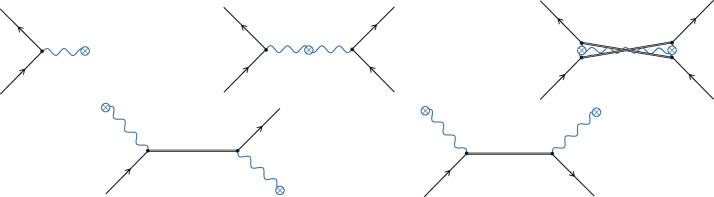
Feynman diagrams for QED with the approximation of classical electromagnetic fields or linear classical gravity. The wiggly blue lines are classical electromagnetic or gravitational fields or potentials. The crosses represent classical sources for the fields or potentials. As in Fig. 1, black lines represent electrons, positrons, or general matter or antimatter particles. For ease of visualization, double lines without arrows represent virtual particles.

The interaction Hamiltonian in equation (4) is analogous to that of an approximation sometimes performed in QED^26,29–31^: for certain QED calculations, such as Rutherford scattering, a good approximation is to ignore the quantumness of the electromagnetic field such that we drop the hat from *A*_*μ*_ in equation (1). Feynman diagrams can be drawn for this theory^26^, where a cross is often used to denote classical electromagnetic potentials and waves or, equivalently, a classical source for the electromagnetic field. For example, Fig. 2 (top left) illustrates an electron interacting with a classical potential or wave, and Fig. 2 (top middle) depicts how two electrons would interact in this theory, where now the cross can be seen as breaking quantum communication channels involving virtual photons in QED. However, certain diagrams still show virtual matter propagating the interactions, such as Fig. 2. (bottom row and top right).

Analogously, we can construct Feynman diagrams from the classical gravity interaction Hamiltonian in equation (4), where now wiggly lines with crosses in Fig. 2 denote classical gravitational potentials and waves. However, just as in the QED approximation, although there are no gravitons, there are still virtual matter propagators (see, for example, Fig. 2, bottom row and top right), and thus quantum communication. Then, as there is quantum communication, classical gravity can create entanglement^2^, violating the theorems and arguments discussed above^4–8^. This occurs because the theorems take a restrictive view on what the gravitational interaction consists of: they essentially assume that quantum gravity involves only virtual graviton propagators, but in fact, at the field theory level, there will also be virtual matter propagators.

## Feynman’s experiment

**Fig. 3:**
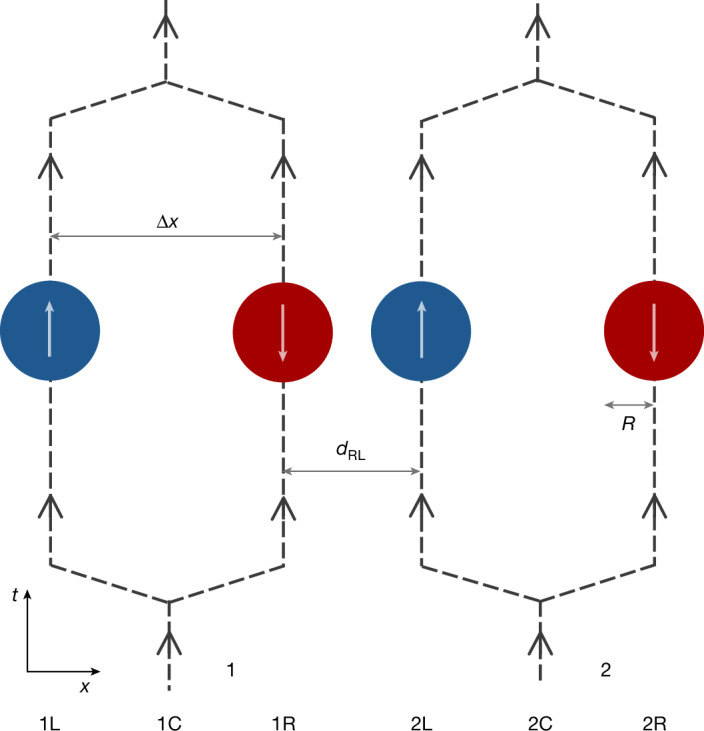
Visualization of a version of Feynman’s experiment. Two spherical mass distributions (1 and 2) of radius *R* are placed in quantum superpositions at two locations as N00N states, with blue and red denoting the components separated by Δ*x*. After gravitationally interacting for a short time, the paths are recombined and entanglement is sought^5,6^. Although Stern–Gerlach interferometry with internal spins is illustrated^5^, alternative set-ups, such as parallel Mach–Zehnders, are also possible^6^. Here, Δ*x* is depicted larger than the minimum separation *d*_RL_, but a general configuration can be implemented, including Δ*x* ≪ *d*_RL_.

To illustrate this further, we now demonstrate how equation (4) leads to entanglement in a version of Feynman’s experiment. Two spherical mass distributions, each with total mass *M* and radius *R*, are prepared in a quantum superposition of two locations. This could be achieved by, for example, implementing matter-wave beam splitters^6^, manipulating potentials^32^ or exploiting internal degrees of freedom, such as quantum spins in Stern–Gerlach experiments^5^ (Fig. 3). Gravity is assumed to be the only interaction between the particles, and in the non-relativistic limit and describing matter within first quantization, it just acts as a quantum phase *φ*_*ij*_ ≔ *GM*^2^*t*/(*ħ* *d*_*ij*_) on each superposition branch^5,6^, where *d*_*ij*_ is the distance between the matter distributions in the branch labelled by *i*, *j* ∈ {L, R}, and *GM*^2^/*d*_*ij*_ is the Newtonian potential energy. With the superposition size Δ*x* much greater than the smallest distance *d*_RL_, only the quantum phase *φ* ≔ *φ*_RL_ is significant, so that the systems are clearly entangled, with the entanglement depending solely on *φ* (refs. ^5,6^). By contrast, when Δ*x* ≪ *d*_RL_, the relevant parameter for entanglement becomes essentially $\overline{\varphi }:= \varphi \,\Delta {x}^{2}/{d}_{{\rm{RL}}}^{2}$ (ref. ^33^). To measure the entanglement, the superposed paths could be recombined and correlations sought between the interferometer outputs^6^ or internal degrees of freedom^5^.

### Quantum gravity

We now analyse this experiment using perturbative quantum gravity with a QFT description of matter. With electromagnetic interactions ignored, we take the initial state of the objects immediately after being placed in a quantum superposition as a product of N00N states:

5 $$\begin{array}{l}|\varPsi \rangle =\frac{1}{2}({|N\rangle }_{{\rm{1L}}}{|0\rangle }_{{\rm{1R}}}{|\uparrow \rangle }_{1}+{|0\rangle }_{{\rm{1L}}}{|N\rangle }_{{\rm{1R}}}{|\downarrow \rangle }_{1})\\ \qquad \otimes ({|N\rangle }_{{\rm{2L}}}{|0\rangle }_{{\rm{2R}}}{|\uparrow \rangle }_{2}+{|0\rangle }_{{\rm{2L}}}{|N\rangle }_{{\rm{2R}}}{|\downarrow \rangle }_{2}),\end{array}$$

where |*N*⟩_*κi*_ is a product of *N* independent position states of matter particles obeying a complex scalar field^34^, with *κ* ∈ {1, 2} and *i* ∈ {L, R} labelling the position of the spherical objects (matching Fig. 3). *N* is the number of particles in the objects, such that *M* = *mN*, with *m* the mass of the particles. We also include possible internal spin states {|↑⟩, |↓⟩}, which could be used to generate the quantum superpositions.

After a time *t*, the state of the matter system in the Schrödinger picture is

6 $$| \varPsi (t)\rangle ={{\rm{e}}}^{{\rm{i}}{\widehat{H}}_{0}t/\hbar }\widehat{T}{{\rm{e}}}^{-({\rm{i}}/\hbar ){\int }_{0}^{t}{\rm{d}}\tau {\widehat{H}}_{{\rm{I}}}(\tau )}| \varPsi \rangle ,$$

where $\widehat{T}$ is the time-ordering operator, *τ* is a dummy time variable, ${\widehat{H}}_{0}$ is the Hamiltonian of perturbative quantum gravity in the absence of matter–gravity interactions and ${\widehat{H}}_{{\rm{I}}}:= \exp ({\rm{i}}{\widehat{H}}_{0}t/\hbar ){\widehat{H}}_{{\rm{int}}}\,\exp (-{\rm{i}}{\widehat{H}}_{0}t/\hbar )$. Just before the superposition paths are bought back together, for example through reverse Stern–Gerlachs^1,5^, we can write

7 $$\begin{array}{l}|\,\varPsi (t)\rangle \propto {\alpha }_{{\rm{LL}}}{|N\rangle }_{{\rm{1L}}}{|N\rangle }_{{\rm{2L}}}+{\alpha }_{{\rm{LR}}}{|N\rangle }_{{\rm{1L}}}{|N\rangle }_{{\rm{2R}}}\\ \qquad \,+\,{\alpha }_{{\rm{RL}}}{|N\rangle }_{{\rm{1R}}}{|N\rangle }_{{\rm{2L}}}+{\alpha }_{{\rm{RR}}}{|N\rangle }_{{\rm{1R}}}{|N\rangle }_{{\rm{2R}}},\end{array}$$

where ${\alpha }_{ij}\in {\mathbb{C}}$. We have now neglected any internal spin states and the vacuum states for simplicity. We can calculate the amplitudes *α*_*ij*_ by taking the inner product of equation (7) (and also equation (6)), with the basis states |*N*⟩_1*i*_ |*N*⟩_2*j*_ and expanding the time-ordered unitary operation in equation (6) as the Dyson series^26,35^. The amplitudes can then be written as a perturbative series with each term corresponding to that of the Dyson series: ${\alpha }_{ij}={\alpha }_{ij}^{(0)}+{\alpha }_{ij}^{(1)}+{\alpha }_{ij}^{(2)}+\cdots \,$. The first process where a virtual graviton is exchanged between the matter objects occurs at second order in the series and corresponds to the Feynman diagram in Fig. 1 (top left). The amplitude for this Feynman diagram, within the very good approximation *ct* ≫ *d*_*ij*_ (Methods), is

8 $$\frac{G{M}^{2}t}{\hbar {V}^{2}}\iint {{\rm{d}}}^{3}{\bf{x}}\,{{\rm{d}}}^{3}{\bf{y}}\frac{{\theta }_{1i}({\bf{x}}){\theta }_{2j}({\bf{y}})}{| {\bf{x}}-{\bf{y}}| }\equiv {\varphi }_{ij},$$

where *θ*_*κi*_(**x**) ≔ *θ*(*R* − ∣**x** − **X**_*κi*_∣) is the unit-step function defining the spherical shape of the matter distribution *κ* in branch *i*, **X**_*κi*_ is the coordinate for the centre of mass for the distributions and *V* ≔ 4π*R*^3^/3. The above amplitude directly contributes to ${\alpha }_{ij}^{(2)}$ such that, when Δ*x* ≫ *d*_RL_, the ${\alpha }_{{\rm{RL}}}^{(2)}$ amplitude dominates over all others and equals i*φ*. Then, given that ${\alpha }_{ij}^{(0)}=1$ from the Dyson series and that ${\alpha }_{ij}^{(1)}$ contains no contractions for the gravitational field, we are left with *α*_*ij*_ ≈ 1 except for *α*_RL_ ≈ 1 + i*φ*, which matches the first-quantized result to first order in the quantum phase $\exp ({\rm{i}}\varphi )$. The full non-perturbative result is straightforwardly obtained by considering the form of the corresponding higher-order Feynman diagrams and extrapolating the result^26^.

### Classical gravity

We now consider the above experiment within the context of classical gravity. The calculation follows the above but with the interaction Hamiltonian of equation (4) rather than equation (2). At second order in the Dyson series there are no non-vanishing Wick contractions corresponding to Feynman diagrams that contain quantum communication between the matter objects, and the diagram responsible for entanglement in quantum gravity, Fig. 1 (top left), becomes Fig. 2 (top middle). This diagram represents the two matter objects sitting in their combined classical gravitational field, with the amplitude just contributing a local relative quantum phase between the branches of each matter object, which does not lead to entanglement^13^. However, at fourth order in the series, a diagram appears where the matter distributions are connected quantum mechanically through virtual matter particles (Fig. 2, top right). Within the same approximations as in the quantum gravity case, the amplitude for the Feynman diagram is (Methods)

9 $${{\vartheta }}_{ij}:= \frac{{m}^{6}{t}^{2}{N}^{2}}{4{{\rm{\pi }}}^{2}{\hbar }^{6}{V}^{2}}{\left(i\int {{\rm{d}}}^{3}{\bf{x}}{{\rm{d}}}^{3}{\bf{y}}\frac{\varPhi ({\bf{x}})\varPhi ({\bf{y}}){\theta }_{1i}({\bf{x}}){\theta }_{2j}({\bf{y}})}{| {\bf{x}}-{\bf{y}}| }\right)}^{2},$$

where *Φ*(**x**) ≔ −*c*^2^*h*^00^(**x**)/2 is the total gravitational potential of the matter objects, and *ϑ*_*ij*_ directly contributes to ${\alpha }_{ij}^{(4)}$ in equation (7). As we have a classical theory of gravity, *Φ*(**x**) is the same in each superposition branch, otherwise the Newtonian force would be in a quantum superposition. Certain works have considered gravity to be classical but still allow the field or Newtonian force to be in a quantum superposition^36–39^. Here, we keep to the notion that quantum superposition is a purely quantum-mechanical phenomena such that *Φ*(**x**) is not superposed in equation (9) and is not a quantum operator. However, despite this, equation (9) generically results in entanglement, as *θ*_1*i*_(**x**) and *θ*_2*i*_(**x**) are different for the different superposition paths and are connected through ∣**x** − **y**∣, such that ${\alpha }_{ij}^{(4)}$ is different for each superposition path, except for ${\alpha }_{{\rm{LL}}}^{(4)}={\alpha }_{{\rm{RR}}}^{(4)}$ from symmetry. We can understand this from the Feynman diagram in Fig. 2 (top right), where, unlike the gravitational potential, the virtual matter particles enter into a quantum superposition with the different mass states and the distance the virtual matter particles propagate is different in each branch, resulting in the spatial integrals in equation (9) being connected through ∣**x** − **y**∣.

As *Φ*(**x**) comes from a superposition of matter in equation (9), we must consider exactly how gravity is sourced by quantum matter in a fundamental theory of classical gravity. In the approach most considered^40,41^, *Φ*(**x**) in equation (9) is the sum of the average potentials of the superposition states of the two objects. In this case, *ϑ*_*ij*_ is inversely proportional to *d*_*ij*_ such that, if Δ*x* ≫ *d*_RL_, the RL amplitude dominates over all others and is ∣*ϑ*_RL_∣ ≈ *ϑ*, where (Methods)

10 $$\sqrt{{\vartheta }}=\frac{6}{25}\frac{{G}^{2}{m}^{2}{M}^{3}Rt}{{\hbar }^{3}{d}_{{\rm{RL}}}}.$$

The state in equation (7) is then clearly entangled, because, just as in quantum gravity, *α*_RL_ contains a contribution *ϑ* that is not in any of the other amplitudes *α*_*ij*_. Furthermore, like quantum gravity^33^, the inverse scaling of *ϑ*_*ij*_ with *d*_*ij*_ allows $\overline{{\vartheta }}:= {\vartheta }\,\Delta {x}^{2}/{d}_{{\rm{RL}}}^{2}$ to be identified as the relevant parameter for entanglement when Δ*x* ≪ *d*_RL_.

## Discussion

The quantum gravity effect, characterized by *φ*, depends strongly on the mass and duration chosen for the experiment. A range of mass values and durations have been considered for Feynman’s experiment^1,5,6,11,42,43^, with the Planck mass, *M*_p_ ≈ 10^−8^ kg, thought to play a significant role^1,32,44^. For example, in ref. ^5^, relatively small masses are suggested, *M* ≈ 10^−14^ kg, at the expense of long coherence times *t* ≈ 2 s and large superpositions Δ*x* ≈ 0.1 mm, and with *d*_RL_ = 200 μm, such that quantum gravity would be an order of magnitude greater than residual electromagnetic interactions (alternatively, a conducting screen can be placed between the objects to eliminate electromagnetic interactions^45^). However, long coherence times present a significant experimental challenge due to expected decoherence mechanisms^33^. For instance, the source of decoherence that is thought to be the most dominant^5^, namely the scattering of molecules from an imperfect vacuum, requires extremely low pressures of 10^−15^ Pa for *M* = 10^−14^ kg and *t* = 2 s, which presents a formidable challenge^5,32,33^. The rate of decoherence from scattering scales linearly with pressure but as *M*^2/3^ with mass, which is far weaker than the mass scaling *M*^2^ of *φ* (ref. ^33^). Therefore, as it allows for much lower pressures, using larger masses and smaller times may be experimentally preferable^33^, which has been considered in equivalent tests^6,46,47^ with times such as 1 μs and masses ranging from 10^−12^ kg to 1 kg (refs. ^1,6,11,33,42,43^). Smaller superposition sizes have also been considered, as creating large Δ*x* has proven experimentally challenging thus far^33^.

**Fig. 4:**
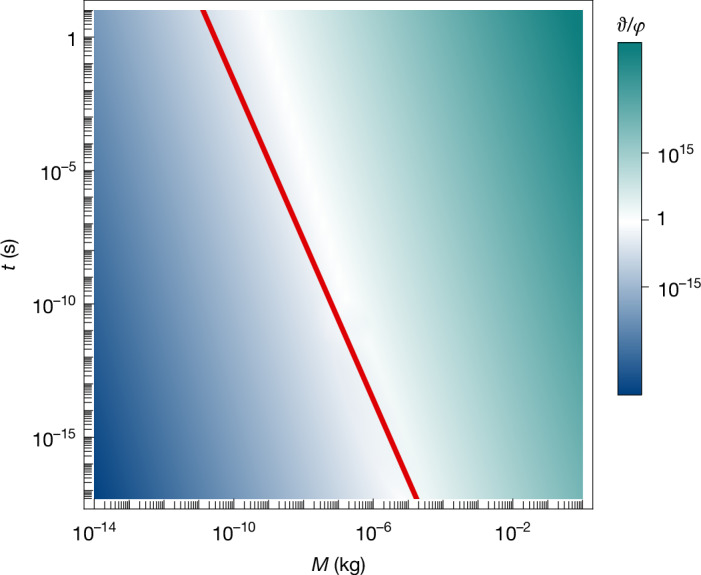
Comparison of classical and quantum gravity effects. The relative strength of the considered effects *ϑ*/*φ* is shown for a range of masses *M* and times *t* in the experiment described in the main text. The red line and the region to the right of the line are where the classical gravity effect and its associated entanglement are significant (*ϑ* ≥ 0.1). To provide evidence of quantum gravity, the experiment must, therefore, operate to the left of this line. A minimum separation *d*_RL_ = 10*R* is assumed, with *R* set by the total mass and density (note that *ϑ* is independent of the density) and *m* the mass of ytterbium. The ratio $\overline{{\vartheta }}/\overline{\varphi }$, which characterizes the relative strength of the effects when *d*_RL_ ≫ Δ*x*, is identical to *ϑ*/*φ*.

The comparative strength of the classical and quantum gravity effects calculated above also depends strongly on the mass and duration of the experiment. Figure 4 compares the classical and quantum gravity effects, *φ* and *ϑ*, for various durations and masses for the experiment described in section ‘Feynman’s experiment’ with ytterbium as the material^5^. For relatively small masses *M* ≈ 10^−14^ kg and long durations *t* ≈ 2 s, *ϑ* is substantially smaller than *φ*. However, with masses approaching the Planck mass and beyond, *ϑ* becomes large enough (*ϑ* ≈ 0.1) such that entanglement due to the classical gravity process is significant, even at short durations. Thus, the mere observation of entanglement at these values in the experiment described above cannot be taken as evidence of quantum gravity.

The classical gravity process that is generating the entanglement is the exchange of virtual quantum matter associated with the gravitational interaction. Entanglement does not, thus, arise from just degrees of freedom that could be purely associated with the classical gravitational field^5–8,48^. As argued above, the very fact that gravity couples to matter implies, when quantum matter is treated within QFT, the possibility of having a matter propagator that can generate entanglement through the gravitational interaction, regardless of the specific form of the classical gravity model. Note that if, for example, electromagnetism were classical and we had electrically charged objects, then a similar equation to equation (9) could be derived with *Φ*(**x**) representing the Coulomb field of the objects. This could also, in principle, lead to entanglement, just as QED provides entanglement through an analogous expression to equation (8) through virtual photon exchange^23,49^. However, unlike with electromagnetism, we do not yet know if gravity is quantum. Here, we have shown that, although entanglement can be used to provide evidence for the quantum nature of gravity, contrary to that considered previously^5–8^, this is not unambiguous and is, instead, fundamentally a phenomenological issue: it depends on the parameters and form of the experiment.

## Methods

The amplitude corresponding to the Feynman diagram in Fig. 1 (top left) can be computed by taking Wick contractions of the form11where  represents the Feynman graviton propagator ${\int }_{t}{{\rm{d}}}^{4}x:= \,\int {{\rm{d}}}^{3}{\bf{x}}{\int }_{0}^{ct}{\rm{d}}{x}^{0}$ and *c* is the speed of light. The above Wick contractions are evaluated using standard QFT techniques^26^, leading to equation (8) in the approximations of the experiment described in the main text and Fig. 3. A pedagogical overview of the calculations is provided in  Supplementary Information.

The Feynman diagram in Fig. 2 (top right) corresponds to Wick contractions of the form12which is evaluated using standard QFT techniques^26^ and results in equation (9) in the approximations of the experiment described in the main text and Fig. 3. A full pedagogical derivation of how the above contraction leads to equation (9) is provided in Supplementary Information, where virtual matter processes in quantum gravity (which are always accompanied by graviton exchange) are also detailed.

As stated in the main text, *Φ*(**x**) in equation (9) is sourced by matter that is in a quantum superposition. The two leading suggestions for how this occurs in a fundamental theory of classical gravity are as follows: (1) Gravity is sourced by the mean expectation of matter ${\nabla }^{2}\varPhi ({\bf{x}})=\xi \langle {\widehat{T}}_{00}\rangle$ (refs. ^40,41^), where *ξ* = 4π*G*/*c*^2^, and the expectation is over the standard quantum state of matter or a generalization, such as a local description^50–52^. (2) Gravity is sourced by stochastic fluctuations around the mean expectation^53–56^: ${\nabla }^{2}\varPhi ({\bf{x}})=\xi [\langle {\widehat{T}}_{00}\rangle +\delta {T}_{00}]$, where *δT*_00_ is a stochastic quantity. The former has been studied more than the latter and is, thus, the option considered in the main text. A discussion on (2) is provided in Supplementary Information. The theoretical consistency of both cases has been debated, as also discussed in Supplementary Information, but neither has been ruled out experimentally. With option (1), *Φ*(**x**) in equation (9) is the sum of the average potentials of each mass distribution over their left and right states:

13 $$\varPhi ({\bf{x}})={\varPhi }_{{\rm{C1}}}({\bf{x}})+{\varPhi }_{{\rm{C2}}}({\bf{x}}),$$

with

14 $${\varPhi }_{{\rm{C}}\kappa }({\bf{x}}):= \frac{1}{2}({\varPhi }_{\kappa {\rm{L}}}({\bf{x}})+{\varPhi }_{\kappa {\rm{R}}}({\bf{x}})),$$

and

15 $$\begin{array}{c}{\varPhi }_{\kappa i}({\bf{x}}):= -\,GM\left[\left(\frac{3}{2R}-\frac{| {\bf{x}}-{{\bf{X}}}_{\kappa i}{| }^{2}}{2{R}^{3}}\right)\theta (R-| {\bf{x}}-{{\bf{X}}}_{\kappa i}| )\right.\\ \,\,\,\,+\left.\frac{\theta (| {\bf{x}}-{{\bf{X}}}_{\kappa i}| -R)}{| {\bf{x}}-{{\bf{X}}}_{\kappa i}| }\right],\end{array}$$

such that *Φ*_*κi*_(**x**) is the gravitational potential of a spherical mass distribution of total mass *M* at position **X**_*κi*_, and *Φ*_C*κ*_(**x**) is the average gravitational potential of spherical mass distributions each of mass *M* located at **X**_*κ*L_ and **X**_*κ*R_. The above potential is then inserted into equation (9), and the amplitude can be computed using standard integration techniques, as demonstrated in Supplementary Information.

## Online content

Any methods, additional references, Nature Portfolio reporting summaries, source data, extended data, supplementary information, acknowledgements, peer review information; details of author contributions and competing interests; and statements of data and code availability are available at 10.1038/s41586-025-09595-7.

## Supplementary information

Supplementary Information Supplementary Sections 1–6, including Supplementary Figs. 1 and 2 and Supplementary references.

## Data Availability

There are no data to be shared.

## Code availability

No generated code was central to the main results. However, Mathematica code used as a guide in calculations can be obtained upon request to the corresponding author.
