# Supplementary material for: Classical theories of gravity produce entanglement
Source: Nature. 2025 Oct 22;646(8086):813–7. doi: 10.1038/s41586-025-09595-7 (PMC12545165; doi:10.1038/s41586-025-09595-7)
Supplement: Supplementary file 1 — Supplementary Sections 1–6, including Supplementary Figs. 1 and 2 and Supplementary references. [file 41586_2025_9595_MOESM1_ESM.pdf]

nature portfolio

https://doi.org/10.1038/s41586-025-09595-7

Supplementary information

Classical theories of gravity produce entanglement

In the format provided by the authors and unedited

Supplementary Information for 'Classical theories of gravity produce entanglement'

Joseph Aziz[1] and Richard Howl[1*]

[1]Department of Physics, Royal Holloway, University of London, Egham, TW20 0EX, Surrey, United Kingdom.

Abstract

This supplementary information provides a pedagogical run-through of the quantum-field calculations for the amplitudes of the Feynman diagrams presented in the main text - Figure 1a (see Section 1) and Figure 2e (see Section 2). It also considers entanglement in theories of stochastic classical gravity (Section 4), discusses the self-consistency of classical gravity theories (Section 5), provides a brief review of the articles mentioned in the main text that consider non-local theories of classical gravity (Section 3), and discuses alternative signatures to entanglement (Section 6).

1 Experiment of main text in linearized quantum gravity

Here, we provide a pedagogical analysis of the experiment discussed in the main text assuming that gravity acts as linearized quantum gravity at low energies.

The full Hamiltonian of the system can be written as

$$\hat{H} = \hat{H}_0 + \hat{H}_{int}, \tag{1}$$

where $\hat{H}_0 := \hat{H}_0^M + \hat{H}_0^G$, with $\hat{H}_0^M$ and $\hat{H}_0^G$ representing respective free Hamiltonians of the matter (complex scalar) and gravitational fields - see e.g. Ref. [1–3] - and $\hat{H}_{int}$ describes the interaction between these two fields - Equation 2 in the main text. As discussed in the main text, gravity is assumed the dominating interaction between the particles in the experiment, and we thus ignore electromagnetic interactions.

We treat linearized quantum gravity as an effective quantum field theory, valid at low energies. The evolution of the quantum state of the system, in the Schrödinger picture, is then given by Equation 6 of the main text, which can be written as

$$|\Psi(t)\rangle = \hat{U}_0^\dagger \hat{U}_I |\Psi\rangle, \tag{2}$$

where $|\Psi\rangle$ is the initial state of the system and

$$\hat{U}_0 := e^{-i\hat{H}_0 t/\hbar}, \tag{3}$$

$$\hat{U}_I := \hat{T} e^{-i\int_0^t d\tau\, \hat{H}_I(\tau)/\hbar}. \tag{4}$$

The unitary operation $\hat{U}_I$ can be expanded as the Dyson series

$$\hat{U}_I = 1 - \frac{i}{\hbar}\int_0^t d\tau\,\hat{H}_I(\tau) - \frac{1}{2\hbar^2}\hat{T}\int_0^t d\tau\,d\tau'\,\hat{H}_I(\tau)\,\hat{H}_I(\tau') + \cdots,$$

where $\hat{H}_I$ is the interaction Hamiltonian in the interaction picture:

$$\hat{H}_I := \hat{U}_0^\dagger \hat{H}_{int}\hat{U}_0 = -\frac{1}{2}\int d^3\boldsymbol{x}\,\hat{h}^{\mu\nu}(x)\hat{T}_{\mu\nu}(x), \tag{5}$$

with $\hat{h}_{\mu\nu}(x)$ the free gravitational field operator in the Heisenberg picture, and $\hat{T}^{\mu\nu}(x)$ is as Equation 3 in the main text but with the free matter field $\hat{\phi}(x)$ in the Heisenberg picture:

$$\hat{\phi}(x) := c\sqrt{\hbar}\int \frac{d^3\boldsymbol{k}}{(2\pi)^3}\frac{1}{\sqrt{2\omega_{\boldsymbol{k}}}}\left(\hat{a}_{\boldsymbol{k}}e^{ik.x} + \hat{b}_{\boldsymbol{k}}^\dagger e^{-ik.x}\right) \tag{6}$$

$$=: \hat{\phi}^{(+)}(x) + \hat{\phi}^{(-)}(x), \tag{7}$$

with $\hat{\phi}^{(+)}(x) := c\sqrt{\hbar}\int d^3\boldsymbol{k}\,\hat{a}_{\boldsymbol{k}}e^{ik.x}/((2\pi)^3\sqrt{2\omega_{\boldsymbol{k}}})$ the positive frequency component of the field; $\hat{\phi}^{(-)}(x) := c\sqrt{\hbar}\int d^3\boldsymbol{k}\,\hat{b}_{\boldsymbol{k}}^\dagger e^{-ik.x}/((2\pi)^3\sqrt{2\omega_{\boldsymbol{k}}})$ the negative frequency component; $k.x := k^\mu x_\mu$; $x^0 = ct$; and $\hat{a}_{\boldsymbol{k}}$ and $\hat{b}_{\boldsymbol{k}}$ are the annihilation operators for matter and antimatter particles respectively, such that $[\hat{a}_{\boldsymbol{k}}, \hat{a}_{\boldsymbol{k'}}^\dagger] = (2\pi)^3\delta^{(3)}(\boldsymbol{k}-\boldsymbol{k'})$, $[\hat{b}_{\boldsymbol{k}}, \hat{b}_{\boldsymbol{k'}}^\dagger] = (2\pi)^3\delta^{(3)}(\boldsymbol{k}-\boldsymbol{k'})$, and $[\hat{a}_{\boldsymbol{k}}, \hat{b}_{\boldsymbol{k'}}^\dagger] = 0$.

The initial state of the system $|\Psi\rangle$ in the experiment is assumed to be Equation 5 of the main text:

$$|\Psi\rangle = \frac{1}{2}\left(|N\rangle_{1L}|0\rangle_{1R}|\uparrow\rangle_1 + |0\rangle_{1L}|N\rangle_{1R}|\downarrow\rangle_1\right)\otimes\left(|N\rangle_{1L}|0\rangle_{2R}|\uparrow\rangle_2 + |0\rangle_{1L}|N\rangle_{2R}|\downarrow\rangle_2\right), \tag{8}$$

where we have included spin degrees of freedom that could be used to generate the spatial superpositions - in this case the above state would be that of the system just after Stern-Gerlach experiments (see also Figure 3 of the main text). In writing $|\Psi\rangle$, we have ignored possible configuration states of the gravitational field and the anti-matter particles since these are not relevant to the following discussion. The state $|N\rangle_{\kappa i}$, with $\kappa \in \{1,2\}$ and $i \in \{L,R\}$, is an N-particle position state [4], which is defined as

$$|N\rangle_{\kappa i} := \frac{1}{\sqrt{N!}}\int \prod_{j=1}^N d^3\boldsymbol{x}_j\tilde{\phi}_{\kappa i}(\boldsymbol{x}_j)|\boldsymbol{x}_j\rangle, \tag{9}$$

where $|\boldsymbol{x}_j\rangle$ is a single-particle position state defined below; $\tilde{\phi}_{\kappa i}(\boldsymbol{x}) := \theta_{\kappa i}(\boldsymbol{x})/\sqrt{V}$, with $\theta_{\kappa i}(\boldsymbol{x}) := \theta(R - |\boldsymbol{x} - \boldsymbol{X}_{\kappa i}|)$ as in the main text; R is the radius of the matter spheres in the experiment; $V = 4\pi R^3/3$; and $\boldsymbol{X}_{\kappa i}$ is the centre-of-mass coordinate for the sphere κ in branch i. This definition of the initial matter state follows from how wavepackets are used to model particles in QFT: in QFT single-particle wavepackets are defined, in general as [1]

$$|\psi\rangle = \int \frac{d^3\boldsymbol{k}}{(2\pi)^3}\frac{1}{\sqrt{2\omega_{\boldsymbol{k}}}}\phi(\boldsymbol{k})|\boldsymbol{k}\rangle, \tag{10}$$

where $|\boldsymbol{k}\rangle := \sqrt{2\omega_{\boldsymbol{k}}}\hat{a}_{\boldsymbol{k}}^\dagger|0\rangle$ and $\phi(\boldsymbol{k})$ is the Fourier transform of the spatial wavefunction $\phi(\boldsymbol{k}) := \int d^3\boldsymbol{x}\tilde{\phi}(\boldsymbol{x})e^{-i\boldsymbol{k}.\boldsymbol{x}}$, with $\int d^3\boldsymbol{x}|\tilde{\phi}(\boldsymbol{x})|^2 = 1$. For a localized particle, its wavepacket $|\boldsymbol{x}\rangle$ is then

$$|\boldsymbol{x}\rangle = \int \frac{d^3\boldsymbol{k}}{(2\pi)^3}\frac{1}{\sqrt{2\omega_{\boldsymbol{k}}}}e^{-i\boldsymbol{k}.\boldsymbol{x}}|\boldsymbol{k}\rangle, \tag{11}$$

and so $\langle\boldsymbol{y}|\boldsymbol{x}\rangle = \delta^{(3)}(\boldsymbol{x}-\boldsymbol{y})$. Using this, we thus define an N-particle wavepacket as (9) with $\tilde{\phi}_{\kappa i}(\boldsymbol{x})$ being the general spatial wavefunction. Taking the atoms as part of a spherical object, then

$\tilde{\phi}_{\kappa i}(\boldsymbol{x}) := \theta_{\kappa i}(\boldsymbol{x})/\sqrt{V}$ as described above, where $\int d^3\boldsymbol{x}|\tilde{\phi}_{\kappa i}(\boldsymbol{x})|^2 = 1$ and also ${}_{\kappa i}\langle N|N\rangle_{\lambda j} = 0$ when the different spheres are non-overlapping $d_{ij} := |\boldsymbol{X}_{\kappa i} - \boldsymbol{X}_{\lambda j}| > 2R$, with $\kappa j \neq \lambda j$, which is satisfied in the experiment. We, therefore, have "orthonormal" initial states, and such states will remain orthonormal under unitary evolution.

After the matter systems have interacted through gravity, we are interested in states of the form:

$$|\Psi(t)\rangle = \frac{1}{\mathcal{N}}\Big(\alpha_{LL}|N\rangle_{1L}|N\rangle_{2L}|\uparrow\rangle_1|\uparrow\rangle_2 + \alpha_{LR}|N\rangle_{1L}|N\rangle_{2R}|\uparrow\rangle_1|\downarrow\rangle_2 + \alpha_{RL}|N\rangle_{1R}|N\rangle_{2L}|\downarrow\rangle_1|\uparrow\rangle_2 + \alpha_{RR}|N\rangle_{1R}|N\rangle_{2R}|\downarrow\rangle_1|\downarrow\rangle_2\Big), \tag{12}$$

where $\mathcal{N}$ is the normalization constant and we have ignored vacuum states for simplicity, as in the main text. This is just the second-quantized, N00N state version of the final state considered in modern interpretations of Feynman's experiment, see e.g. [5, 6].[1] After the interaction, the matter systems are brought back together and interfered [5, 6]. For example, in the case of embedded spins, reverse Stern-Gerlach devices could be used such that the above state becomes

$$|\Psi(t)\rangle = \frac{1}{\mathcal{N}}\left(\alpha_{LL}|\uparrow\rangle_1|\uparrow\rangle_2 + \alpha_{LR}|\uparrow\rangle_1|\downarrow\rangle_2 + \alpha_{RL}|\downarrow\rangle_1|\uparrow\rangle_2 + \alpha_{RR}|\downarrow\rangle_1|\downarrow\rangle_2\right) \otimes |N\rangle_{1C}|N\rangle_{2C}, \tag{13}$$

where κC is the position of the matter object κ [5]. As described in the proposal [5], the spins of the particles can be measured to determine whether the matter systems are entangled. This entanglement is clearly only dependent on the values that the amplitudes $\alpha_{LL}, \alpha_{LR}, \alpha_{RL}$ and α_{RR} take, and since the amplitudes in (13) are the same as in (12), we can just use (12) in calculating them. We do this through the standard perturbative QFT technique of acting the expected final states on the evolved initial states, such that

$$\alpha_{ij} = {}_{1i}\langle N|\,{}_{2j}\langle N|\hat{U}_0^\dagger\hat{U}_I|\Psi\rangle = {}_{1i}\langle N|\,{}_{2j}\langle N|\hat{U}_0^\dagger\hat{U}_I|N\rangle_{1i}|N\rangle_{2j}, \tag{14}$$

where we have used the orthonormality property of $|N\rangle_{\kappa i}$. As we show below, see (24), the unitary operator $\hat{U}_0^\dagger$ just acts the same phase on each superposition branch so that it only provides a global phase on the full state. This is due to the fact that, within the approximations of the experiment, the objects stay fixed under free evolution. We can, therefore, ignore the action of $\hat{U}_0$ and just concentrate on $\hat{U}_I$, which matches the original experimental proposal, where the free evolution is also ignored [5]. Considering that $\hat{U}_I$ can be expanded in the Dyson series above, we write α_{ij} as $\alpha_{ij} = \alpha_{ij}^{(0)} + \alpha_{ij}^{(1)} + \alpha_{ij}^{(2)} + \cdots$, where $\alpha_{ij}^{(n)}$ (with $n \in \{0, 1, 2, \ldots\}$) corresponds to the particular order of the Dyson series. From (4), at zeroth order there is just unity and so $\alpha_{ij}^{(0)} = 1$ for all i, j, which is analogous to the trivial part of the usual S-matrix [1]. At first order in (4), there are no corresponding Feynman diagrams that involve virtual gravitons such that we can ignore this order, which we detail further below. However, at second order, we have

$$\alpha_{ij}^{(2)} = -\frac{1}{2\hbar^2}\,{}_{1i}\langle N|\,{}_{2j}\langle N|\hat{T}\int_0^t d\tau d\tau' \hat{H}_I(\tau)\hat{H}_I(\tau')|N\rangle_{1i}|N\rangle_{2j}, \tag{15}$$

$$= -\frac{1}{8\hbar^2 c^2}\,{}_{1i}\langle N|\,{}_{2j}\langle N|\hat{T}\int_t d^4x\, d^4y\, \hat{h}^{\mu\nu}(x)\hat{T}_{\mu\nu}(x)\hat{h}^{\rho\sigma}(y)\hat{T}_{\rho\sigma}(y)|N\rangle_{1i}|N\rangle_{2j}, \tag{16}$$

[1]Note that in the non-relativistic limit, by itself, $\hat{H}_I$ would in principle also allow states of the form $|N-k\rangle_{1i}|N+k\rangle_{2j}$ and $|N+k\rangle_{1i}|N-k\rangle_{2j}$, where k is some integer. These states would not be seen in the experiment (see discussion at end of Section 1) and, even if they were, would only contribute to entanglement since there can be no final states of the form $|N-k\rangle_{1i}|N-k\rangle_{2j}$ or $|N+k\rangle_{1i}|N+k\rangle_{2j}$, since the total particle number is conserved.

where $\int_t d^4x := \int_0^{ct} dx^0 \int d^3\boldsymbol{x}$. The above can be computed using Wick contractions as per standard QFT [1]. Following Equation 11 in the Methods, the relevant contractions are of the form:

$$\gamma_{ij}^{(2)} := -\frac{1}{4\hbar^2 c^2}\int_t d^4x \int_t d^4y \times$$
$$ {}_{1i}\langle N|\ {}_{2j}\langle N|\hat{\mathcal{T}}_{\mu\nu}[\hat{\phi}^\dagger(x)\hat{\phi}(x)]\hat{\mathcal{T}}_{\rho\sigma}[\hat{\phi}^\dagger(y)\hat{\phi}(y)]\hat{h}^{\mu\nu}(x)\hat{h}^{\rho\sigma}(y)|N\rangle_{1i}\,|N\rangle_{2j}, \tag{17}$$

where $\hat{\mathcal{T}}_{\mu\nu}$ is defined in Equation 3 in the main text, and a factor of two has been included due to symmetry (we can contract $\hat{\phi}(x)$ with either $|N\rangle_{1i}$ or $|N\rangle_{2j}$). This contraction essentially corresponds to the Feynman diagram 1a in the main text. All other Wick contractions either correspond to unconnected bubble diagrams, which we can ignore [1], or do not contribute to entanglement. Of note is the contraction corresponding to Feynman diagram 1a, which we show below gives a vanishing contribution in the approximation we are working, since it involves an atom in one object moving to the other object. Note that, although we refer to the drawn diagrams as 'Feynman diagrams', they should be seen more as a visualization of the process in an analogous way to standard Feynman diagrams rather than strictly proper Feynman diagrams. This is because we are assuming position-like in and out states - Equation (9) - and so the momentum of the external legs can be zero. The arrow on the external legs should then be considered as representing a flow in time more than a flow in space.

In (17), the contraction of the gravitational fields is the graviton Feynman propagator. In the Lorenz gauge, this is [7, 8]:

$$\hat{h}^{\mu\nu}(x)\hat{h}^{\rho\sigma}(y) = \frac{16\pi G\hbar}{c^3}\left(\eta^{\mu\rho}\eta^{\nu\sigma} + \eta^{\mu\sigma}\eta^{\nu\rho} - \eta^{\mu\nu}\eta^{\rho\sigma}\right)\int \frac{d^4k}{(2\pi)^4}\frac{-i}{k^2 - i\epsilon}e^{ik.(x-y)}, \tag{18}$$

which leaves the contraction of the matter field on our "in" and "out" states. Following the usual definition of contracting matter fields on in and out momentum states [1], the contraction on position states is:

$$\hat{\phi}(x)|N\rangle_{\kappa i} = \frac{1}{\sqrt{N!}}\int \prod_{j=1}^{N} d^3\boldsymbol{x}_j \tilde{\phi}_{\kappa i}(\boldsymbol{x}_j)\hat{\phi}^{(+)}(x)|\boldsymbol{x}_j\rangle \tag{19}$$

$$= c\sqrt{\hbar}\frac{1}{\sqrt{N!}}\int \prod_{j=1}^{N} d^3\boldsymbol{x}_j \tilde{\phi}_{\kappa i}(\boldsymbol{x}_j)\int \frac{d^3\boldsymbol{k}}{(2\pi)^3}\frac{1}{\sqrt{2\omega_{\boldsymbol{k}}}}e^{ik.x}\hat{a}_{\boldsymbol{k}}|\boldsymbol{x}_j\rangle \tag{20}$$

$$= c\sqrt{\hbar}\sqrt{N}\int d^3\boldsymbol{y}\,\tilde{\phi}_{\kappa i}(\boldsymbol{y})\int \frac{d^3\boldsymbol{k}}{(2\pi)^3}\frac{1}{\sqrt{2\omega_{\boldsymbol{k}}}}e^{ik_0x^0}e^{i\boldsymbol{k}.(\boldsymbol{y}-\boldsymbol{x})}|N-1\rangle_{\kappa i} \tag{21}$$

$$= c\sqrt{\hbar}\sqrt{N}\int \frac{d^3\boldsymbol{k}}{(2\pi)^3}\frac{1}{\sqrt{2\omega_{\boldsymbol{k}}}}e^{ik.x}\tilde{\phi}_{\kappa i}(\boldsymbol{k})|N-1\rangle_{\kappa i}, \tag{22}$$

where $\tilde{\phi}_{\kappa i}(\boldsymbol{k}) := \int d^3\boldsymbol{x}e^{-\boldsymbol{k}.\boldsymbol{x}}\tilde{\phi}_{\kappa i}(\boldsymbol{x})$ is the Fourier transform of $\tilde{\phi}_{\kappa i}(\boldsymbol{x})$. Given that $\tilde{\phi}_{\kappa i}(\boldsymbol{x}) := \theta_{\kappa i}(\boldsymbol{x})/\sqrt{V}$, its Fourier transform is $\tilde{\phi}_{\kappa i}(\boldsymbol{k}) = 4\pi\left(\sin(|\boldsymbol{k}|R) - |\boldsymbol{k}|R\cos(|\boldsymbol{k}|R)\right)/\sqrt{V}|\boldsymbol{k}|^3$. As long as $R \gg \hbar/(mc)$, which we would expect in a realistic experiment, then $\tilde{\phi}_{\kappa i}(\boldsymbol{k})$ rapidly drops off as $|\boldsymbol{k}|$ increases and is approximately zero before $|\boldsymbol{k}|$ gets close to $mc/\hbar$. This all follows from the fact that the in and out states we have chosen, (9), are *non-relativistic* as long as $R \gg \hbar/(mc)$. In this non-relativistic approximation, since $\tilde{\phi}_{\kappa i}(\boldsymbol{k})$ is almost vanishing before $|\boldsymbol{k}| \approx mc/\hbar$, we can approximate ω_k in (21) with $mc^2/\hbar$, which follows the usual non-relativistic definition of in and out momentum states used in standard perturbative QFT calculations, such as the derivation of the Coulomb potential [1]. This then results in

$$\hat{\phi}(x)|N\rangle_{\kappa i} \approx \frac{\hbar}{\sqrt{2m}}\sqrt{N}e^{-imcx^0/\hbar}\tilde{\phi}_{\kappa i}(\boldsymbol{x})|N-1\rangle_{\kappa i}, \tag{23}$$

where the factor $e^{-imcx^0/\hbar}\tilde{\phi}_{\kappa i}(\boldsymbol{x})$ comes from the fact that we have essentially assumed stationary, single-particle matter waves for the in and out matter states. In the same approximation, the free unitary operator $\hat{U}_0(t) = \hat{U}_0^G(t)\exp(-i\int d^3\boldsymbol{k}\,\omega_k \hat{a}^\dagger_{\boldsymbol{k}}\hat{a}_{\boldsymbol{k}} t/(2\pi)^3)$ in (14) acts on the final state a global phase $2Mc^2t/\hbar$, where $\hat{U}_0^G(t)$ is free evolution associated with the gravitational field [2, 3]:

$$
\begin{aligned}
&\int \frac{d^3\boldsymbol{p}}{(2\pi)^3}\hbar\omega_{\boldsymbol{p}}\hat{a}^\dagger_{\boldsymbol{p}}\hat{a}_{\boldsymbol{p}}|N\rangle_{1i},|N\rangle_{2j}\\
&= \frac{1}{N!}\int \frac{d^3\boldsymbol{p}}{(2\pi)^3}\prod_s^N d^3\boldsymbol{x}_s\, d^3\boldsymbol{y}_s\tilde{\phi}_{1i}(\boldsymbol{x}_s)\tilde{\phi}_{2j}(\boldsymbol{y}_s)\hbar\omega_{\boldsymbol{p}}\hat{a}^\dagger_{\boldsymbol{p}}\hat{a}_{\boldsymbol{p}}|\boldsymbol{x}_s\rangle|\boldsymbol{y}_s\rangle\\
&= \frac{1}{N!}\int \prod_s^N d^3\boldsymbol{x}_s\, d^3\boldsymbol{y}_s \frac{d^3\boldsymbol{k}_s\, d^3\boldsymbol{q}_s\, d^3\boldsymbol{p}}{(2\pi)^{3(1+2s)}}\tilde{\phi}_{1i}(\boldsymbol{x}_s)\tilde{\phi}_{2j}(\boldsymbol{y}_s)e^{-i\boldsymbol{k}_s\cdot\boldsymbol{x}_s}e^{-i\boldsymbol{q}_s\cdot\boldsymbol{y}_s}\hbar\omega_{\boldsymbol{p}}\hat{a}^\dagger_{\boldsymbol{p}}\hat{a}_{\boldsymbol{p}}\hat{a}^\dagger_{\boldsymbol{k}_s}\hat{a}^\dagger_{\boldsymbol{q}_s}|0\rangle\\
&= \frac{1}{N!}\int\left(\prod_s^N d^3\boldsymbol{x}_s\, d^3\boldsymbol{y}_s\frac{d^3\boldsymbol{k}_s\, d^3\boldsymbol{q}_s}{(2\pi)^{6s}}\tilde{\phi}_{1i}(\boldsymbol{x}_s)\tilde{\phi}_{2j}(\boldsymbol{y}_s)e^{-i\boldsymbol{k}_s\cdot\boldsymbol{x}_s}e^{-i\boldsymbol{q}_s\cdot\boldsymbol{y}_s}\hat{a}^\dagger_{\boldsymbol{k}_s}\hat{a}^\dagger_{\boldsymbol{q}_s}\right)\sum_t^N\hbar(\omega_{\boldsymbol{k}_t}+\omega_{\boldsymbol{q}_t})|0\rangle\\
&= \frac{1}{N!}\int\frac{d^3\boldsymbol{k}}{(2\pi)^3}\hbar\omega_{\boldsymbol{k}}\hat{a}^\dagger_{\boldsymbol{k}}\sum_n^N\left(\prod_{s\neq n}^{N-1}\prod_t^N\int d^3\boldsymbol{x}_n\tilde{\phi}_{1i}(\boldsymbol{x}_n)e^{-i\boldsymbol{k}\cdot\boldsymbol{x}_n}+\prod_s^N\prod_{t\neq n}^{N-1}\int d^3\boldsymbol{y}_n\tilde{\phi}_{2j}(\boldsymbol{y}_n)e^{-i\boldsymbol{k}\cdot\boldsymbol{y}_n}\right)\\
&\qquad\qquad\times\int d^3\boldsymbol{x}_s\, d^3\boldsymbol{y}_t\,\tilde{\phi}_{1i}(\boldsymbol{x}_s)\tilde{\phi}_{2j}(\boldsymbol{y}_t)|\boldsymbol{x}_s\rangle|\boldsymbol{y}_t\rangle\\
&= \frac{N}{N!}\int\frac{d^3\boldsymbol{k}}{(2\pi)^3}\hbar\omega_{\boldsymbol{k}}\hat{a}^\dagger_{\boldsymbol{k}}\left(\prod_{s\neq n}^{N-1}\prod_t^N\tilde{\phi}_{1i}(\boldsymbol{k})+\prod_s^N\prod_{t\neq n}^{N-1}\tilde{\phi}_{2j}(\boldsymbol{k})\right)\int d^3\boldsymbol{x}_s\, d^3\boldsymbol{y}_t\,\tilde{\phi}_{1i}(\boldsymbol{x}_s)\tilde{\phi}_{2j}(\boldsymbol{y}_t)|\boldsymbol{x}_s\rangle|\boldsymbol{y}_t\rangle\\
&\approx \frac{Nmc^2}{N!}\int\frac{d^3\boldsymbol{k}}{(2\pi)^3}\hat{a}^\dagger_{\boldsymbol{k}}\left(\prod_{s\neq n}^{N-1}\prod_t^N\tilde{\phi}_{1i}(\boldsymbol{k})+\prod_s^N\prod_{t\neq n}^{N-1}\tilde{\phi}_{2j}(\boldsymbol{k})\right)\int d^3\boldsymbol{x}_s\, d^3\boldsymbol{y}_t\,\tilde{\phi}_{1i}(\boldsymbol{x}_s)\tilde{\phi}_{2j}(\boldsymbol{y}_t)|\boldsymbol{x}_s\rangle|\boldsymbol{y}_t\rangle\\
&= \frac{Nmc^2}{N!}\int\frac{d^3\boldsymbol{k}}{(2\pi)^3}\hat{a}^\dagger_{\boldsymbol{k}}\sum_n^N\left(\prod_{s\neq n}^{N-1}\prod_t^N\int d^3\boldsymbol{x}_n\tilde{\phi}_{1i}(\boldsymbol{x}_n)e^{-i\boldsymbol{k}\cdot\boldsymbol{x}_n}+\prod_s^N\prod_{t\neq n}^{N-1}\int d^3\boldsymbol{y}_n\tilde{\phi}_{2j}(\boldsymbol{y}_n)e^{-i\boldsymbol{k}\cdot\boldsymbol{y}_n}\right)\\
&\qquad\qquad\times\int d^3\boldsymbol{x}_s\, d^3\boldsymbol{y}_t\,\tilde{\phi}_{1i}(\boldsymbol{x}_s)\tilde{\phi}_{2j}(\boldsymbol{y}_t)|\boldsymbol{x}_s\rangle|\boldsymbol{y}_t\rangle\\
&= \frac{2Mc^2}{N!}\prod_s^N\int d^3\boldsymbol{x}_s d^3\boldsymbol{y}_s\tilde{\phi}_{1i}(\boldsymbol{x}_s)\tilde{\phi}_{2j}(\boldsymbol{y}_s)|\boldsymbol{x}_s\rangle|\boldsymbol{y}_s\rangle\\
&= 2Mc^2|N\rangle_{1i},|N\rangle_{2j}\\
&\implies \exp(-i\int d^3\boldsymbol{k}\,\omega_k\hat{a}^\dagger_{\boldsymbol{k}}\hat{a}_{\boldsymbol{k}}t/(2\pi)^3)|N\rangle_{1i},|N\rangle_{2j}\approx e^{2Mic^2t/\hbar}|N\rangle_{1i},|N\rangle_{2j}.
\end{aligned}
\tag{24}
$$

Since the free evolution by itself just contributes a global phase, it can be ignored. That is, within the approximations of the experiment, the objects stay fixed under free evolution.

In addition to the simple contraction $\overbracket{\hat{\phi}(x)|N\rangle}_{\kappa i}$, we also have contractions of derivatives of the field coming from the energy-momentum tensor operator $\hat{\mathcal{T}}_{\mu\nu}$. For example, $\partial_\mu\overbracket{\hat{\phi}(x)|N\rangle}_{\kappa i}$. However, given the above non-relativistic approximation, the only relevant terms in this case are those where the derivative is in the time coordinate: $\partial_0\overbracket{\hat{\phi}(x)|N\rangle}_{\kappa i}$, which is just the time derivative of the right-hand side of (23) in our non-relativistic approximation.

With the above contraction (23), we are operating in a low-energy regime suitable to adequately describe the experiment, which also justifies the use of linearized quantum gravity as an effective field theory. Using this contraction, (23), with the gravitational contraction (18), we can now compute the

amplitude $\gamma_{ij}^{(2)}$. This leaves us with

$$\gamma_{ij}^{(2)} = \frac{4i\pi GM^2}{\hbar c} \int_t d^4x \int_t d^4y \int \frac{d^4k}{(2\pi)^4} \frac{1}{k^2} e^{ik.(x-y)} \, |\tilde{\phi}_{1i}(\boldsymbol{x})|^2 |\tilde{\phi}_{2j}(\boldsymbol{y})|^2. \tag{25}$$

We first integrate over $\boldsymbol{k}$:

$$\int \frac{d^3\boldsymbol{k}}{(2\pi)^3} \frac{1}{-(k^0)^2 + \boldsymbol{k}^2} e^{i\boldsymbol{k}.(\boldsymbol{x}-\boldsymbol{y})} = \frac{1}{4\pi} \frac{1}{|\boldsymbol{x}-\boldsymbol{y}|} e^{-ik^0|\boldsymbol{x}-\boldsymbol{y}|}. \tag{26}$$

Next we integrate over k^0:

$$\frac{1}{4\pi|\boldsymbol{x}-\boldsymbol{y}|} \int \frac{dk^0}{2\pi} e^{-ik^0[(|\boldsymbol{x}-\boldsymbol{y}|-(x^0-y^0)]} = \frac{1}{4\pi|\boldsymbol{x}-\boldsymbol{y}|} \delta^{(3)}(|\boldsymbol{x}-\boldsymbol{y}| - (x^0-y^0)), \tag{27}$$

and then we can integrate over x^0 and y^0 from 0 to ct, finding

$$\begin{aligned} &\frac{1}{4\pi|\boldsymbol{x}-\boldsymbol{y}|} \int_0^{ct} \int_0^{ct} dx^0 dy^0 \delta^{(3)}(|\boldsymbol{x}-\boldsymbol{y}| - (x^0-y^0)) \\ &\qquad = \frac{1}{4\pi} \left(\frac{ct}{|\boldsymbol{x}-\boldsymbol{y}|} - 1 \right) \theta(ct - |\boldsymbol{x}-\boldsymbol{y}|). \end{aligned} \tag{28}$$

Plugging this back into (25), we have

$$\gamma_{ij}^{(2)} = \frac{iGM^2}{\hbar c} \int d^3\boldsymbol{x} \int d^3\boldsymbol{y} |\tilde{\phi}_{1i}(\boldsymbol{x})|^2 |\tilde{\phi}_{2j}(\boldsymbol{y})|^2 \left(\frac{ct}{|\boldsymbol{x}-\boldsymbol{y}|} - 1 \right) \theta(ct - |\boldsymbol{x}-\boldsymbol{y}|) \tag{29}$$

$$= \frac{iGM^2}{\hbar c V^2} \int d^3\boldsymbol{x} \int d^3\boldsymbol{y} \, \theta_{1i}(\boldsymbol{x}) \, \theta_{2j}(\boldsymbol{y}) \left(\frac{ct}{|\boldsymbol{x}-\boldsymbol{y}|} - 1 \right) \theta(ct - |\boldsymbol{x}-\boldsymbol{y}|). \tag{30}$$

This is the *relativistic* expression (taking into account the finite speed of gravity) of the quantum phases for each superposition branch. It upgrades the relativistic expression derived in Ref. [9] from non-relativistic point particles to spherical objects. We can re-derive the expression for point particles using the approximation $d_{ij} \gg R$ (for example, moving the $\boldsymbol{x}$ and $\boldsymbol{y}$ coordinates to the centres of the respective spheres and using $d_{ij} \gg R$, noting the integration bounds of the new coordinates when considering the integration of $|\boldsymbol{x}-\boldsymbol{y}|$), which results in:

$$\gamma_{ij}^{(2)} \approx \frac{iGM^2}{\hbar c} \left(\frac{ct}{d_{ij}} - 1 \right) \theta(ct - d_{ij}). \tag{31}$$

As discussed in Ref. [9], this demonstrates that entanglement between the initially spacelike separated matter objects, which we have assumed here, is only generated once the initial light cone of one object contains the other object. This is because it takes a finite time for the information of one object's position to be delivered to the other (through the mediating virtual gravitons), respecting causality.

Finally, when $ct \gg d_{ij}$, which is very much going to be the case for a realistic experiment, we arrive at the originally derived, fully non-relativistic version of the quantum phases [5, 6] $\gamma_{ij}^{(2)} = i\varphi_{ij} := iGM^2 t/(\hbar d_{ij})$, where $d_{ij} := |\boldsymbol{X}_{1i} - \boldsymbol{X}_{2j}|$. We could have also got here immediately after using (26) and then assuming $ct \gg 1$, since after (26) we could have first performed the time integrals rather than the k^0 integral:

$$\begin{aligned} \gamma_{ij}^{(2)} = \frac{iGM^2}{\hbar c} \int d^3\boldsymbol{x} \int d^3\boldsymbol{y} \frac{\tilde{\phi}_{1i}^2(\boldsymbol{x}) \tilde{\phi}_{2j}^2(\boldsymbol{y})}{|\boldsymbol{x}-\boldsymbol{y}|} \times \\ \int_0^{ct} dx^0 e^{ix^0k_0} \int_0^{ct} dy^0 e^{-iy^0k_0} \int \frac{dk^0}{2\pi} e^{-ik^0|\boldsymbol{x}-\boldsymbol{y}|} \end{aligned} \tag{32}$$

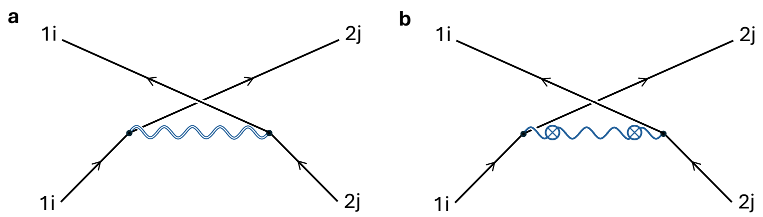

Fig. 1 a) Feynman diagram corresponding to Wick contraction (37). The $1i$ and $2j$ label the first and second matter distributions, with $i, j \in \{L, R\}$. b) The corresponding diagram when there is a classical gravity interaction (the two circles with crosses indicate the two classical sources of gravity, i.e. the two matter distributions). The amplitudes of both diagrams are found to be vanishing. In contrast to standard pertubative QFT diagrams, the external legs here represent position states rather than definite momentum states, as detailed in the main supplementary text, with the arrows indicating time evolution. In the case of definite momentum, the corresponding Feynman diagrams are, in general, non-vanishing, as is well-known [1].

$$= \frac{iGM^2}{\hbar c} \int d^3\boldsymbol{x} \int d^3\boldsymbol{y} \frac{\tilde{\phi}^2_{1i}(\boldsymbol{x})\tilde{\phi}^2_{2j}(\boldsymbol{y})}{|\boldsymbol{x}-\boldsymbol{y}|} \times \int dk^0 \int_0^{ct} dy^0 e^{-iy^0k_0} e^{-ik^0|\boldsymbol{x}-\boldsymbol{y}|} e^{ictk_0/2} \frac{\sin(ctk_0/2)}{\pi k_0} \tag{33}$$

$$\approx \frac{iGM^2}{\hbar c} \int d^3\boldsymbol{x} \int d^3\boldsymbol{y} \frac{\tilde{\phi}^2_{1i}(\boldsymbol{x})\tilde{\phi}^2_{2j}(\boldsymbol{y})}{|\boldsymbol{x}-\boldsymbol{y}|} \times \int dk^0 \int_0^{ct} dy^0 e^{-iy^0k_0} e^{-ik^0|\boldsymbol{x}-\boldsymbol{y}|} e^{ictk_0/2} \delta(k_0) \tag{34}$$

$$= \frac{iGM^2}{\hbar c V^2} \int d^3\boldsymbol{x} \int d^3\boldsymbol{y} \frac{\theta_{1i}(\boldsymbol{x})\,\theta_{2j}(\boldsymbol{y})}{|\boldsymbol{x}-\boldsymbol{y}|} \int_0^{ct} dy^0 = i\varphi_{ij}, \tag{35}$$

where we have used $\lim_{\gamma\to\infty} \sin(\gamma x)/(\pi x) = \delta(x)$.

Ignoring the other Wick contraction (Figure 1a) and the first order contributions for now, as well as any second-order contributions that just result in a global phase,[2] when considering the full amplitudes $\alpha_{ij} \approx \alpha_{ij}^{(0)} + \alpha_{ij}^{(2)}$, we have $\alpha_{ij} \approx 1 + i\varphi_{ij}$, which is just the first order expansion of the quantum phase $e^{i\varphi_{ij}}$. Therefore, with $d_{RL} \gg \Delta x$, as assumed in the main text, the final state in the Stern-Gerlach version of the experiment would be of the form:

$$|\Psi(t)\rangle = \frac{1}{2}\left(|\uparrow\rangle_1|\uparrow\rangle_2 + |\uparrow\rangle_1|\downarrow\rangle_2 + (1+i\varphi)|\downarrow\rangle_1|\uparrow\rangle_2 + |\downarrow\rangle_1|\downarrow\rangle_2\right), \tag{36}$$

to second order in the Dyson series, which is an entangled state. As this is a perturbative calculation, where we have ignored terms higher than second order in the Dyson series, the result (36) is only valid for $\varphi := \varphi_{RL} \ll 1$ and is just the first-order expansion of the quantum phase $e^{i\varphi}$ that was previously derived in first-quantization works [5, 10] - also see the main text. However, the full non-perturbative expression $e^{i\varphi}$ can straightforwardly be obtained by considering that at each even order in the Dyson series we have essentially the Feynman diagram 1a of the main text again but with an extra graviton propagator and additional in and out states. The amplitude $\gamma_{ij}^{(2)}$ is then just taken to an extra power together with the corresponding factorial from the Dyson series, providing the Taylor expansion of $e^{i\varphi}$. It is common in perturbative QFT that a low-order calculation can be extrapolated to a non-perturbative result [1].

With $\Delta x \ll d_{RL}$, each phase φ_{ij} is approximately the same and, rather than entanglement only depending on φ, the relevant parameter is now $\varphi\, \Delta x^2/d_{RL}^2$ as discussed in the main text. This can be derived by considering the overlap of the superposition states [11] or, for example, considering the expression for the negativity of the final state [12].

We now further discuss the first order Dyson series terms and Feynman diagram Figure 1a. At first order, there is no way to internally Wick contract the gravitational field, so this order does not

[2]Such as gravitational self-interactions of the matter objects.

contribute to the process we are interested in; while for the second-order Feynman diagram 1a, the corresponding contraction is:

$$-\frac{1}{4\hbar^2c^2}\int_t d^4x\int_t d^4y\ {}_{1i}\langle N|\ {}_{2j}\langle N|\hat{\mathcal{T}}_{\mu\nu}[\hat{\phi}^\dagger(x)\hat{\phi}(x)]\hat{h}^{\mu\nu}(x)\hat{h}^{\rho\sigma}(y)\hat{\mathcal{T}}_{\rho\sigma}[\hat{\phi}^\dagger(y)\hat{\phi}(y)]|N\rangle_{1i}\,|N\rangle_{2j}. \tag{37}$$

From (23), this amplitude contains the integrals:

$$\int d^3\boldsymbol{x}\int d^3\boldsymbol{y}\,\tilde{\phi}_{1j}(\boldsymbol{x})\tilde{\phi}_{2j}(\boldsymbol{x})\tilde{\phi}_{1j}(\boldsymbol{y})\tilde{\phi}_{2j}(\boldsymbol{y}). \tag{38}$$

Since the wavefunctions do not overlap, as described above, this integral evaluates to zero. The Feynman diagram thus provides a vanishing amplitude. This is because it is not possible for one atom of one object to freely diffuse from one object to the other. That is, we assume that there are no "direct" interactions between the two matter systems and, as such, only contractions of the form (16) (Figure 1a in the main text) contribute to entanglement at this order in the Dyson series. Therefore, before second order we have a separable state, and find a non-separable state (36) at second order. Note that separability cannot be 'repaired' by going to a higher (and thus numerically weaker) order in the expansion - the fact that we see non-separability of the state at second order is enough to demonstrate that the full state must be entangled, and as discussed above, this is just due to the second order expansion of the quantum phase $e^{i\varphi}$.

2 Experiment of main text in linearized classical gravity

We now provide a full analysis of the experiment discussed in the main text assuming that gravity acts as classical gravity. To achieve this, we apply the same methodology and steps outlined above when considering quantum gravity but now use the interaction Hamiltonian Equation 4 in the main text rather than Equation 2.

As in the previous section, we write the full Hamiltonian as:

$$\hat{H} = \hat{H}_0 + \hat{H}_{int}, \tag{39}$$

where $\hat{H}_0$ describes the free evolution and $\hat{H}_{int}$ describes the interaction, which is now between quantum matter and *classical* gravity - Equation 4 in the main text. The free part is again split into gravitational and matter components: $\hat{H}_0 = \hat{H}_0^M + H_0^G$, but now there are no quantum gravitational field degrees of freedom and so H_G^0 is just a classical Hamiltonian (see e.g. Ref. [2, 3, 7]).

The evolution of the quantum state of the system follows that provided in the previous section (2), and the Dyson series is similarly expanded as below (4). The initial and final system states ("in" and "out" states) are also taken to be (8) and (13), and we calculate the amplitudes in the final state by the same procedure of acting the final state on the evolved initial state (12). Just as in the above quantum gravity section, the two matter objects $|N\rangle_{1i}$ and $|N\rangle_{2j}$ are prepared spacelike separated: $\tilde{\phi}_{\kappa i}(\boldsymbol{x}) = \theta_{\kappa i}/\sqrt{V}$, with $d_{ij} := |\boldsymbol{X}_{1i} - \boldsymbol{X}_{2j}|$, and are held fixed in position modes for the duration of the experiment we are interested in.

The second-order process (17) (Figure 1a in the main text) that resulted in entanglement in the previous section, now takes the form:

$$\beta_{ij}^{(2)} = -\frac{1}{8\hbar^2c^2}\int_t d^4x\int_t d^4y\, h^{\mu\nu}(x)\, h^{\rho\sigma}(y)\times \\ {}_{1i}\langle N|\ {}_{2j}\langle N|\hat{\mathcal{T}}_{\mu\nu}[\hat{\phi}^\dagger(x)\hat{\phi}(x)]\hat{\mathcal{T}}_{\rho\sigma}[\hat{\phi}^\dagger(y)\hat{\phi}(y)]|N\rangle_{1i}\,|N\rangle_{2j}, \tag{40}$$

and corresponds to diagram Figure 2b in the main text (also Figure 2c here). Now $h_{\mu\nu}(x)$ is the classical gravitational field of the matter objects (and satisfying $|h_{\mu\nu}| \ll \eta_{\mu\nu}$ as discussed in the main

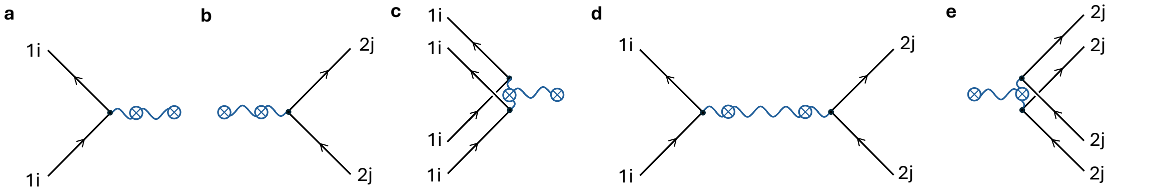

Fig. 2 First and second order Feynman diagrams that contribute towards the relative quantum phases $\Delta\varphi_1$ and $\Delta\varphi_2$ in (50) but not entanglement. The $1i$ and $2j$ label the first and second matter distributions, with $i, j \in \{L, R\}$. The two circles with crosses indicate the two classical sources of gravity, i.e. the two matter distributions. As stated also in Figure 1, in contrast to standard perturbative QFT diagrams, the external legs represent position states, as detailed in the main supplementary text, with the arrows indicating time evolution.

text and $h_{\mu\nu} \to 0$ as $|\boldsymbol{x}| \to \infty$), and so, since it is classical, there is no Wick contraction for it (there is no associated non-commutativity). Crucially, since it is not associated with a quantum operator, it takes the *same* value in each superposition branch. If this were not the case, then the gravitational field would be in a quantum superposition, and thus not classical. This has caused confusion in the literature with works, such as [13–16], that consider gravity classical but still allow the field or the Newtonian force to go into a superposition such that (40) can result in the same amplitude and thus entanglement as (16) in the previous section (see below for more detail). We do not assume such a scenario here, keeping to the notion that quantum superposition is a purely quantum-mechanical phenomena.

As in the previous section, in computing (40) we can consider a non-relativistic approximation for the in and out states (see (23)), such that we only need consider contractions of the field and its time derivative on the in and out states, Equation (23). As in the quantum gravity case, this means that the free evolution generated by $\hat{H}_0$ only contributes a global phase and thus no entanglement - see Equation (24). That is, we can ignore the free evolution generated by $\hat{H}_0$, just as in the original experimental proposal [5]. The objects after being prepared spacelike separated, thus remain fixed in position modes. Furthermore, since we know that non-relativistic gravity is a good approximation for the experiment, we can further assume that $h_{\mu\nu}(\boldsymbol{x}) = -2\Phi(\boldsymbol{x})\delta_{\mu\nu}/c^2$, with $\Phi(\boldsymbol{x})$ the Newtonian potential of the matter objects, which is assumed spatially varying and time-independent. In this approximation, the interaction Hamiltonian simplifies to:

$$\hat{H}_{int} = \frac{4}{c^2}\int d^3\boldsymbol{x}\,\Phi(\boldsymbol{x})\Big(\hat{\pi}(\boldsymbol{x})\hat{\pi}^\dagger(\boldsymbol{x}) - \frac{m^2c^2}{2\hbar^2}\hat{\phi}^\dagger(\boldsymbol{x})\hat{\phi}(\boldsymbol{x})\Big), \tag{41}$$

which in the interaction picture is simply

$$\hat{H}_I = \frac{4}{c^2}\int d^3\boldsymbol{x}\,\Phi(\boldsymbol{x})\Big(\hat{\pi}(x)\hat{\pi}^\dagger(x) - \frac{m^2c^2}{2\hbar^2}\hat{\phi}^\dagger(x)\hat{\phi}(x)\Big), \tag{42}$$

where $\hat{\pi} := \partial_0\hat{\phi}^\dagger$. Now we can immediately see that we only have to worry about the contraction of the field and its time-derivative on the in and out states, rather than any spatial derivatives, such as $\partial_x\overbracket{\hat{\phi}(x)|N}\rangle_{\kappa i}$. Using $\overbracket{\hat{\pi}^\dagger(x)|N}\rangle_{\kappa i} \approx -i\sqrt{mN/2}ce^{-imcx^0/\hbar}\tilde{\phi}_{\kappa i}(\boldsymbol{x})|N-1\rangle_{\kappa i}$, we find:

$$\beta^{(2)}_{ij} = -\varphi_{1i}\varphi_{2j}, \tag{43}$$

where:

$$\varphi_{\kappa i} = \frac{Mt}{\hbar}\int d^3\boldsymbol{x}\,|\tilde{\phi}_{\kappa i}(\boldsymbol{x})|^2\Phi(\boldsymbol{x}) \tag{44}$$

$$= \frac{Mt}{\hbar V} \int d^3\boldsymbol{x}\, \theta_{\kappa i}(\boldsymbol{x}) \Phi(\boldsymbol{x}), \tag{45}$$

with $i, j \in \{L, R\}$. Since the two matter objects are no longer quantum-mechanically connected, the amplitude (40) does not contribute to entanglement. Instead, it combines with the first-order and second-order processes in Figure 2 to generate the amplitudes

$$\alpha_{ij} = \alpha_{ij}^{(0)} + \alpha_{ij}^{(1)} + \alpha_{ij}^{(2)} = 1 - i\left(\varphi_{1i} + \varphi_{2j}\right) - \frac{1}{2}\left(\varphi_{1i} + \varphi_{2j}\right)^2, \tag{46}$$

to second order, such that the final state would be

$$|\Psi(t)\rangle = \frac{1}{2}\Bigg(\left[|N\rangle_{1L} \left(1 + i\varphi_{1L} - \frac{1}{2}\varphi_{1L}^2\right) + |N\rangle_{1R} \left(1 + i\varphi_{1R} - \frac{1}{2}\varphi_{1R}^2\right)\right] \tag{47}$$

$$\otimes \left[|N\rangle_{2L} \left(1 + i\varphi_{2L} - \frac{1}{2}\varphi_{2L}^2\right) + |N\rangle_{2R} \left(1 + i\varphi_{2R} - \frac{1}{2}\varphi_{2R}^2\right)\right] \Bigg), \tag{48}$$

which is the second-order approximation to

$$|\Psi(t)\rangle = \frac{1}{2}\Bigg(\left(e^{i\varphi_{1L}}|N\rangle_{1L} + e^{i\varphi_{1R}}|N\rangle_{1R}\right) \otimes \left(e^{i\varphi_{2L}}|N\rangle_{2L} + e^{i\varphi_{2R}}|N\rangle_{2R}\right) \Bigg) \tag{49}$$

$$\equiv \frac{1}{2}\Bigg(\left(|N\rangle_{1L} + e^{i\Delta\varphi_1}|N\rangle_{1R}\right) \otimes \left(|N\rangle_{2L} + e^{i\Delta\varphi_2}|N\rangle_{2R}\right) \Bigg), \tag{50}$$

with $\Delta\varphi_1 := \varphi_{1R} - \varphi_{1L}$ and $\Delta\varphi_2 := \varphi_{2R} - \varphi_{2L}$. This is just the state of two matter spheres in a quantum superposition of two locations sitting in an external classical gravitational potential. In traditional semi-classical gravity [17, 18], $\Phi(\boldsymbol{x})$ is sourced by the expectation of the quantum matter objects:

$$\Phi(\boldsymbol{x}) = -\frac{G}{c^2} \int d^3\boldsymbol{y} \frac{\left\langle \psi \left| \hat{T}_{00}(\boldsymbol{y}) \right| \psi \right\rangle}{|\boldsymbol{x} - \boldsymbol{y}|}, \tag{51}$$

where $|\psi\rangle$ is the joint quantum state of the matter objects. This results in $\Phi(\boldsymbol{x})$ being the sum of the average potentials of each mass over their left and right states - see Equation 13 to 15 in Methods, which are reproduced below (also see Section 5.1 for more detail):

$$\Phi(\boldsymbol{x}) = \Phi_{C1}(\boldsymbol{x}) + \Phi_{C2}(\boldsymbol{x}), \tag{52}$$

with

$$\Phi_{C\kappa}(\boldsymbol{x}) := \frac{1}{2}\left(\Phi_{\kappa L}(\boldsymbol{x}) + \Phi_{\kappa R}(\boldsymbol{x})\right), \tag{53}$$

and

$$\Phi_{\kappa i}(\boldsymbol{x}) := -GM\left[\left(\frac{3}{2R} - \frac{|\boldsymbol{x} - \boldsymbol{X}_{\kappa i}|^2}{2R^3}\right) \theta(R - |\boldsymbol{x} - \boldsymbol{X}_{\kappa i}|) + \frac{\theta(|\boldsymbol{x} - \boldsymbol{X}_{\kappa i}| - R)}{|\boldsymbol{x} - \boldsymbol{X}_{\kappa i}|}\right], \tag{54}$$

such that $\Phi_{\kappa i}(\boldsymbol{x})$ is the gravitational potential of a spherical mass M at position $\boldsymbol{X}_{\kappa i}$, and $\Phi_{C\kappa}(\boldsymbol{x})$ is the average gravitational potential of the spherical mass distributions each of total mass M located at $\boldsymbol{X}_{\kappa L}$ and $\boldsymbol{X}_{\kappa R}$. Using this, results in the separable state that has been derived previously for semi-classical gravity in the first-quantized picture [19]. Note that the same $\Phi(\boldsymbol{x})$ (52) results if we chose to perform the expectation of $\hat{T}_{00}$ with the 'local' state of matter [20, 21] or chose a relativistic collapse

mechanism [22], as discussed further in Sections 5 and 5.1. The results of this and the next section are thus independent on whether traditional semi-classical gravity is used or these modified ‘local’ versions. Also note that the classical analogue of Figure 1a, which is provided in Figure 1b, is still vanishing since an atom cannot freely diffuse from one object to another.

In addition to contractions (40), at second order there are also contractions involving virtual matter propagators within each object (not between the objects):

$$\int_t d^4x\, d^4y\ {}_{1i}\langle N|\,{}_{2j}\langle N|\Phi(\boldsymbol{y})\Phi(\boldsymbol{x})\hat{\mathcal{T}}[\hat{\phi}^\dagger(y)\hat{\phi}(y)]\hat{\mathcal{T}}[\hat{\phi}^\dagger(x)\hat{\phi}(x)]|N\rangle_{1i}\,|N\rangle_{2j}$$
$$+\int_t d^4x\, d^4y\ {}_{1i}\langle N|\,{}_{2j}\langle N|\Phi(\boldsymbol{y})\Phi(\boldsymbol{x})\hat{\mathcal{T}}[\hat{\phi}^\dagger(y)\hat{\phi}(y)]\hat{\mathcal{T}}[\hat{\phi}^\dagger(x)\hat{\phi}(x)]|N\rangle_{1i}\,|N\rangle_{2j} =: \delta_{1j}+\delta_{2j}. \tag{55}$$

These also just contribute a local relative phase and thus no entanglement: for the different superposition branches ij, the overall amplitude due to this process up to second order is $\alpha^{(2)}_{ij}\approx 1+\delta_{1i}+\delta_{2j}$, which is just that expected from a product state (up to order second order). That is:

$$|\psi\rangle \propto \alpha^{(2)}_{LL}|N\rangle_{1L}|N\rangle_{2L}+\alpha^{(2)}_{LR}|N\rangle_{1L}|N\rangle_{2R}+\alpha^{(2)}_{RL}|N\rangle_{1R}|N\rangle_{2L}+\alpha^{(2)}_{RR}|N\rangle_{1R}|N\rangle_{2R} \tag{56}$$
$$\propto (1+\delta_{1L}+\delta_{2L})|N\rangle_{1L}|N\rangle_{2L}+(1+\delta_{1L}+\delta_{2R})|N\rangle_{1L}|N\rangle_{2R} \tag{57}$$
$$+(1+\delta_{1R}+\delta_{2L})|N\rangle_{1R}|N\rangle_{2L}+(1+\delta_{1R}+\delta_{2R})|N\rangle_{1R}|N\rangle_{2R}, \tag{58}$$

which we can write (to order $\delta_{\kappa i}$) as

$$|\psi\rangle \propto [(1+\delta_{1L})|N\rangle_{1L}+(1+\delta_{1R})|N\rangle_{1R}]\otimes[(1+\delta_{2L})|N\rangle_{2L}+(1+\delta_{2R}|N\rangle_{2R})]\,, \tag{59}$$

where the amplitudes $\delta_{\kappa i}$ collect an i from the virtual matter propagator ((63) below) so that they are just first order contributions of $e^{i\delta_{\kappa i}}$. From geometry, $\delta_{1L}=\delta_{2R}$ and $\delta_{1R}=\delta_{2L}$, so this process just contributes

$$|\psi\rangle \propto \left(|N\rangle_{1L}+e^{i\theta}|N\rangle_{1R}\right)\otimes\left(|N\rangle_{2L}+e^{-i\theta}|N\rangle_{2R}\right), \tag{60}$$

where $i\theta := \delta_{2L}-\delta_{2R}$. This process then, by itself, does not contribute towards entanglement.

2.1 Entanglement

We have seen above that at second order the two objects are in a separable state. This is because there is no possibility to generate a quantum propagator between the two objects up to this order. The same applies also at third order, as can be simply deduced by considering the possible connected Feynman diagrams at this order. Instead, we have to move to the fourth order for there to be a quantum propagator between the objects, and the corresponding diagram for this process is Figure 2e in the main text. Before taking the non-relativistic gravity limit, the amplitude for this diagram takes the form (see also Equation 12 in Methods):

$$\beta^{(4)}_{ij}=\frac{1}{16\,\hbar^4c^4}\int_t d^4x\int_t d^4y\int_t d^4z\int_t d^4w\, h^{\mu\nu}(w)\,h^{\rho\sigma}(z)\,h^{\gamma\delta}(y)\,h^{\kappa\lambda}(x)\times$$
$${}_{1i}\langle N|\,{}_{2j}\langle N|\hat{\mathcal{T}}_{\mu\nu}[\hat{\phi}^\dagger(w)\hat{\phi}(w)]\hat{\mathcal{T}}_{\rho\sigma}[\hat{\phi}^\dagger(z)\hat{\phi}(z)]\hat{\mathcal{T}}_{\gamma\delta}[\hat{\phi}^\dagger(y)\hat{\phi}(y)]\hat{\mathcal{T}}_{\kappa\lambda}[\hat{\phi}^\dagger(x)\hat{\phi}(x)]|N\rangle_{1i}\,|N\rangle_{2j}. \tag{61}$$

With the non-relativistic approximation (42), the amplitude involving just the field $\hat{\phi}$ and not the momentum conjugate $\hat{\pi}$ is

$$\frac{4m^6N^2}{\hbar^6c^2}\int_t d^4x\int_t d^4y\int_t d^4w\int_t d^4z\int\frac{d^4k}{(2\pi)^4}\frac{1}{k^2+m^2c^2/\hbar^2}e^{ik.(x-z)}\times$$

$$\int \frac{d^4l}{(2\pi)^4}\frac{1}{l^2+m^2c^2/\hbar^2}e^{il.(w-y)}\tilde{\phi}_{2j}(\boldsymbol{x})\tilde{\phi}_{1i}(\boldsymbol{z})\tilde{\phi}_{1i}(\boldsymbol{y})\tilde{\phi}_{2j}(\boldsymbol{w})\times$$
$$\Phi(\boldsymbol{x})\Phi(\boldsymbol{y})\Phi(\boldsymbol{z})\Phi(\boldsymbol{w})e^{imc(z^0-x^0)/\hbar}e^{imc(y^0-w^0)/\hbar}, \tag{62}$$

where the integrals over k and l are coming from the virtual matter Feynman propagator for scalar fields [1]:

$$\overbrace{\hat{\phi}(x)\hat{\phi}^\dagger}(y) = c\hbar\int \frac{d^4k}{(2\pi)^4}\frac{i}{k^2+m^2c^2/\hbar^2+i\epsilon}e^{ik.(x-y)}. \tag{63}$$

The contractions also involving the momentum conjugate $\hat{\pi}$ give the expression (61) above but with time derivatives on the phase factors $\exp(imcx^0/\hbar)$ (due to contractions of $\hat{\pi}$ with the in or out states), and/or the Feynman propagators.

Following the previous section, we first integrate over $\boldsymbol{k}$ (and $\boldsymbol{l}$):[3]

$$\int \frac{d^3\boldsymbol{k}}{(2\pi)^3}\frac{1}{\boldsymbol{k}^2-(k^0)^2+\gamma^2}e^{i\boldsymbol{k}.(\boldsymbol{x}-\boldsymbol{y})} = \frac{1}{4\pi^2 ir}\int_{-\infty}^{\infty}dk\frac{ke^{ikr}}{k^2+\tilde{\gamma}^2}, \tag{64}$$

where $k := |\boldsymbol{k}|$, $\gamma := mc/\hbar$, $\tilde{\gamma}^2 := \gamma^2 - k_0^2$ and $r := |\boldsymbol{x}-\boldsymbol{y}|$. The integral has poles at $k = \pm i\tilde{\gamma}$, such that:

$$\frac{1}{4\pi^2 ir}\int_{-\infty}^{\infty}dk\frac{ke^{ikr}}{k^2+\tilde{\gamma}^2} = f(k_0) := \frac{1}{4\pi r}\Bigg(\theta(k_0^2-\gamma^2)\left(e^{-r\sqrt{\gamma^2-k_0^2}}-1\right)$$
$$+\theta(\gamma^2-k_0^2)\left(\cos(r\sqrt{k_0^2-\gamma^2})-1\right)+1\Bigg). \tag{65}$$

Note that when $\gamma = 0$, as in the previous section, we obtain (26).

We now follow the second method used in the above quantum gravity section in obtaining the expression for $\gamma_{ij}^{(2)}$: we perform the time integrals and take a delta function approximation such that:

$$\int_0^{ct}dx^0\int_0^{ct}dz^0 e^{i\gamma(z^0-x^0)}\int\frac{dk^0}{2\pi}f(k_0)e^{ik_0(x^0-z^0)} \tag{66}$$
$$= \int_0^{ct}dz^0\int\frac{dk^0}{2\pi}f(k_0)e^{iz^0(\gamma-k_0)}e^{ict(k_0-\gamma)/2}\frac{\sin(ct(k_0-\gamma)/2)}{k_0-\gamma} \tag{67}$$
$$\approx \int_0^{ct}dz^0\int du\, e^{ictu/2}e^{-iz^0u}\delta(u)f(u+\gamma) \tag{68}$$
$$= \frac{ct}{4\pi r}, \tag{69}$$

where we have used $u := k_0 - \gamma$. Often interaction processes involving massive virtual particles come with exponential decay factors over space [1], as in the first term of (65). However, this is not the case for the process considered here. This is because the contraction of the matter field with the position-like states – see (23) – provides a factor $e^{i\gamma(z^0-x^0)}$ in (66). Without this factor, the time integral sets $k_0 = 0$, resulting in the evaluation of (65) but with $k_0 = 0$, and thus exponential decay with space. In contrast, with the factor $e^{i\gamma(z^0-x^0)}$, we get $k^0 = \gamma$ rather than $k^0 = 0$, resulting in no exponential decay factors over space. However, despite no exponential decay with space, the corresponding particles are still virtual particles as they are in general off-shell (we are integrating over all momenta in (64)), which is further discussed in Section 2.3 from a physical perspective. Note that by taking the above delta function approximation, we are essentially performing a non-relativistic approximation, just as we did through equations (32)-(35) in the quantum gravity calculation of Section 1. Since this is

[3] Here, $\Phi(\boldsymbol{x})$ is considered sufficiently spatially varying in the experiment, see (52), that we do not apply a high-momentum cut-off as in (22). That is, $\int d^3\boldsymbol{x}\, e^{i\boldsymbol{k}.\boldsymbol{x}}\tilde{\phi}(\boldsymbol{x})\Phi(\boldsymbol{x}) \not\approx \tilde{\phi}(\boldsymbol{k})$.

non-relativistic, the interaction then looks like a direct, non-local interaction in this approximation. However, just as for the quantum gravity case, this is just an approximation taken for computational ease, and the actual physical process is local and due to virtual matter exchange (similar to virtual graviton exchange in the quantum gravity case) - see Figure 2e in the main text. This is equivalent to how, for convenience, we might simplify certain calculations in fully classical gravity by taking the non-relativistic limit of general relativity, yet really the physical process that we are attempting to describe is local [9, 23, 24].

For the contractions where the momentum conjugate is also used in the virtual propagator, i.e. $\hat{\pi}(x)\hat{\phi}(y)$ and $\hat{\pi}(x)\hat{\pi}^{\dagger}(y)$, the time derivatives result in an extra factor of $i\gamma$ and γ^2 receptively. Using (69) in (61), we then obtain Equation 9 from the main text:

$$\beta_{ij}^{(4)} \approx \frac{m^6 t^2 N^2}{4\pi^2 \hbar^6 V^2} \left(i \int d^3\boldsymbol{x} \int d^3\boldsymbol{y} \frac{\Phi(\boldsymbol{x})\,\Phi(\boldsymbol{y})\,\theta_{1i}(\boldsymbol{x})\,\theta_{2j}(\boldsymbol{y})}{|\boldsymbol{x}-\boldsymbol{y}|} \right)^2 . \tag{70}$$

As stated above, $\Phi(\boldsymbol{x})$ is the *same* irrespective of i and j - it is the same for each superposition branch since it is a classical potential. Despite this, and in contrast to the classical gravity amplitude (46), $\beta_{ij}^{(4)}$ will, in general, be different for each superposition branch because the object functions $\theta_{1i}(\boldsymbol{x})$ and $\theta_{2j}(\boldsymbol{y})$ are connected through the term $|\boldsymbol{x}-\boldsymbol{y}|$ in the denominator. This is analogous to the quantum gravity expression for $\alpha_{ij}^{(2)}$ in (16) - there the linking denominator came from the virtual graviton, whereas here it comes from virtual matter. That is, although $\Phi(\boldsymbol{x})$ does not quantum superpose, the virtual matter particles do and the distance they must travel in each superposition branch is different just as is the case for virtual gravitons in $\alpha_{ij}^{(2)}$ in quantum gravity. This then leads to a different amplitude for each branch.

In semi-classical gravity [17, 18], $\Phi(\boldsymbol{x})$ is given by (52). Plugging (52) into (70), we are integrating all the different gravitational potentials over the different superposition branches. We solve these integrals by integrating first over $\boldsymbol{y}$ and then over $\boldsymbol{x}$ using a well-known technique for finding the gravitational potential of an axially symmetric mass distribution:

$$\varPhi(x', \theta_x) := \int d^3\boldsymbol{y'}\, \frac{\rho(y', \theta_y)}{|\boldsymbol{y'} - \boldsymbol{x'}|}, \tag{71}$$

which can be written as

$$\varPhi(x', \theta_x) = \sum_{n=0}^{\infty} \varPhi_n(x') P_n(\cos\theta_x), \tag{72}$$

where

$$\varPhi_n(x') = -\frac{2\pi}{(n+1/2)x'^{(n+1)}} \int_0^{x'} dy'\, y'^{(n+2)} \rho_n(y') - \frac{2\pi x'^{n}}{n+1/2} \int_{x'}^{\infty} dy'\, y'^{(1-n)} \rho_n(y'). \tag{73}$$

For example, using the above, we can solve integrals such as

$$I = \int d^3\boldsymbol{x} \int d^3\boldsymbol{y}\, \frac{1}{|\boldsymbol{x}-\boldsymbol{X}_{1L}|\,|\boldsymbol{y}-\boldsymbol{x}|\,|\boldsymbol{y}-\boldsymbol{X}_{1R}|} \theta(R-|\boldsymbol{x}-\boldsymbol{X}_{1R}|)\theta(R-|\boldsymbol{y}-\boldsymbol{X}_{2R}|) \tag{74}$$

$$= \int d^3\boldsymbol{x} \frac{\theta(R-|\boldsymbol{x'}-\boldsymbol{\Delta_1}|)}{x'} \int d^3\boldsymbol{y'} \frac{1}{|\boldsymbol{y'}-\boldsymbol{d_{RR}}|} \frac{1}{|\boldsymbol{y'}-\boldsymbol{x'}|} \theta(R-y'), \tag{75}$$

where $\boldsymbol{y'} := \boldsymbol{y} - \boldsymbol{X}_{1R}$, $\boldsymbol{x'} = \boldsymbol{x} - \boldsymbol{X}_{1R}$, $\boldsymbol{d_{RR}} = \boldsymbol{X}_{1R} - \boldsymbol{X}_{2R}$ and $\boldsymbol{\Delta_1} = \boldsymbol{X}_{1R} - \boldsymbol{X}_{1L}$. We then choose the coordinate system $\boldsymbol{y'}$ such that its z-direction is along $\boldsymbol{d_{RR}}$. In this case

$$\frac{1}{|\boldsymbol{y'}-\boldsymbol{d_{RR}}|} \equiv \frac{1}{d_{RR}\sqrt{1 - 2\frac{y'}{d_{RR}} + \frac{y'^2}{d_{RR}}}} = \frac{1}{d_{RR}} \sum_{m=0}^{\infty} P_m(\cos\theta_y) \left(\frac{y'}{d_{RR}}\right)^m , \tag{76}$$

where $d_{RR} = |\boldsymbol{d_{RR}}|$, $P_m(x)$ is the Legendre polynomials, and θ_y is the polar angle of the $\boldsymbol{y'}$ coordinate system. We can then write I as

$$I = \int d^3\boldsymbol{x'} \frac{\theta(R - |\boldsymbol{x'} - \boldsymbol{\Delta_1}|)}{x'} \int d^3\boldsymbol{y'} \frac{\rho(y', \theta_y)}{|\boldsymbol{y'} - \boldsymbol{x'}|}, \tag{77}$$

where

$$\rho(y', \theta_y) := \frac{\theta(R - y')}{d_{RR}} \sum_{m=0}^{\infty} P_m(\cos\theta_y) \left(\frac{y'}{d_{RR}}\right)^m . \tag{78}$$

We can again now use the above solution of the gravitational potential of an axially symmetric mass distribution. In this case, $\rho_n(y') := (n+1/2)\int_0^\pi \rho(y', \theta_y) P_n(\cos\theta_y)\sin\theta_y d\theta_y = \theta(R-x')x'^n/d_{RR}^{n+1}$ using the orthogonal property of the Legendre polynomials: $\int_{-1}^{1} P_n(x)P_m(x)dx = \delta_{nm}/(n+1/2)$. Plugging our $\rho_n(y')$ into (73), we find

$$\begin{aligned}\Phi_n(x') = \frac{2\pi}{(n+1/2)d_{RR}^{n+1}} \Bigg[\left(\frac{R^2 x'^n}{2} - \frac{x'^{(n+2)}(n+1/2)}{2n+3} \right) \theta(R - x') \\ + \left(\frac{R^{2n+3}}{x'^{(n+1)}(2n+3)} \right) \theta(x' - R) \Bigg].\end{aligned} \tag{79}$$

Inserting this into I, only the second term survives in the assumption that the sphere states do not overlap ($\Delta x > 2R$), leaving us with

$$I = \sum_{n=0}^{\infty} \frac{2\pi R^{2n+3}}{(n+1/2)(2n+3)d_{RR}^{n+1}} \int d^3\boldsymbol{x'} \frac{P_n(\cos\theta_x)}{x'^{(n+2)}} \theta(R - |\boldsymbol{x'} - \boldsymbol{\Delta_1}|). \tag{80}$$

We now assume $d_{RR} \gg R$. In this case, we only need to consider the $n = 0$ term:

$$I \approx \frac{4\pi R^3}{3d_{RR}} \int d^3\boldsymbol{x'} \frac{1}{x'^2} \theta(R - |\boldsymbol{x'} - \boldsymbol{\Delta_1}|). \tag{81}$$

This can then be solved by integrating over the sphere at $\boldsymbol{\Delta_1}$ from the origin of the $\boldsymbol{x'}$ coordinate system with surfaces of constant radius [25]:

$$\begin{aligned} I &\approx \frac{4\pi R^3}{3d_{RR}} \int_0^{2\pi} \int_{\Delta x - R}^{\Delta x + R} \int_0^{cos^{-1}((r^2 + (\Delta x)^2 - R^2)/(2r\Delta x))} \sin\theta \, dr \, d\theta \, d\phi && (82) \\ &= \frac{8\pi^2 R^3}{3d_{RR}} \left(R + \frac{1}{2\Delta x} \left(R^2 - (\Delta x)^2\right) \ln(\frac{\Delta x + R}{\Delta x - R}) \right). && (83) \end{aligned}$$

Applying this integration method to (70) with $\Phi(\boldsymbol{x})$ given by (52), it is possible to solve all the spatial integrals. Then, in the approximations $\Delta x \gg R$ and $d_{ij} \gg R\ \forall i, j$ (which match the approximations used in deriving the final quantum gravity result (35)), $\beta_{ij}^{(4)}$ is found to be:

$$\beta_{ij}^{(4)} \approx \left(\frac{6}{25} \frac{iG^2 m^2 M^3 R t}{\hbar^3 d_{ij}} \right)^2 . \tag{84}$$

Just as with the quantum gravity amplitude $\alpha_{ij}^{(2)}$, there is an inverse dependence on d_{ij}. Therefore, with $d_{RL} \ll \Delta x$, the $\beta_{RL}^{(4)}$ amplitude, which results in Equation 10 in the main text, dominates over

all other $\beta_{ij}^{(4)}$ amplitudes clearly illustrating that the quantum state is *entangled.*[4] As noted above, this is because the matter objects are connected through virtual matter, which can enter a quantum superposition with the matter branches, requiring it to travel different distances in each branch.

With $\Delta x \ll d_{RL}$, just as for quantum gravity, the amplitudes $\beta_{ij}^{(4)}$ become similar and the parameter relevant to entanglement becomes $\beta_{RL}^{(4)} \Delta x^2/d_{RL}^2$. As with quantum gravity, this can be derived by considering the overlap of the superposition states [11], or considering, for example, the negativity of the final state [12].

Note that in addition to $\beta_{ij}^{(4)}$ contributing to α_{ij} in the final state (13), there will also be contributions, up to fourth order, from the processes considered in the previous section. However, as shown in the previous section, these contributions can be written as relative phases between the objects (see (50) and (60)) and can thus be derived by considering local unitaries acting on the initial state (even though, for example, they will depend, like $\beta_{RL}^{(4)}$, explicitly on d_{RL}). Therefore, although these relative phases between the objects contribute to the amplitudes α_{ij}, they do not contribute to the entanglement of the final state.

2.2 Linearity

We first considered the virtual matter process in this section within QFT in curved spacetime. This is the theory used, for example, by Hawking to derive Hawking radiation of a black hole and is considered the appropriate limit of quantum gravity when the field can be approximated classical. It should also, therefore, be a limit of theories of fundamentally classical gravity. However, in QFT in curved spacetime, we do not specify how gravity is sourced by quantum matter, which is required to estimate the size of the entanglement effect in the experiment. Up to Equation (70), we assume that gravity is sourced by the quantum matter of the experiment but do not specify exactly how except that this must satisfy the assumptions of the experiment, such as $\beta_{ij}^{(4)}$ is small enough for the perturbative approach in deriving it to be valid. After this, we specialized to semi-classical gravity [17, 18, 21, 22], the most well-known theory of fundamental classical gravity. In this theory, the gravitational field is sourced by the expectation of the energy-momentum tensor of matter, which results in $\Phi(\boldsymbol{x})$ following (52) in the Newtonian regime. The quantum state of matter $|\psi\rangle$ then evolves according to the Schrödinger equation:

$$
\begin{aligned}
i\hbar\frac{d|\psi\rangle}{dt} &= \hat{H}|\psi\rangle & (85)\\
&= \left(\hat{H}_0 + \hat{H}_{int}\right)|\psi\rangle, & (86)
\end{aligned}
$$

where $\hat{H}_{int}$ in the regime we are interested in for the experiment is (41).

Since $\Phi(\boldsymbol{x})$ now depends on the quantum state of matter, Equation (86) is, in general, non-linear, which is thought to introduce superluminal signalling unless the theory is modified further, as discussed in Section 5. However, due to the experimental design where the matter objects are held in fixed position states using magnetic fields, and because we work to a perturbative order, we operate in a regime where the theory is *linear* as in standard quantum mechanics. To see this, we first note that $\Phi(\boldsymbol{x})$ can be written in general as:

$$\Phi[\psi(t)](\boldsymbol{x}) = G\tilde{\Phi}[\psi(t)](\boldsymbol{x}), \tag{87}$$

where:

$$\tilde{\Phi}[\psi(t)](\boldsymbol{x}) := -\frac{1}{c^2}\int d^3\boldsymbol{x}'\,\frac{\langle\psi(t)|\hat{T}_{00}(\boldsymbol{x}')|\psi(t)\rangle}{|\boldsymbol{x}-\boldsymbol{x}'|}. \tag{88}$$

[4]Note that unlike with the classical gravity amplitude $\beta_{ij}^{(2)}$ above, there are no other Feynman diagram amplitudes at fourth order or lower for $\beta_{RL}^{(4)}$ to combine with to make a separable state.

The interaction Hamiltonian can then be written as $\hat{H}_{int} = G\hat{\tilde{H}}_{int}$, with

$$\hat{\tilde{H}}_{int} := \frac{4}{c^2}\int d^3\boldsymbol{x}\tilde{\Phi}[\psi(t)](\boldsymbol{x})\left(\hat{\pi}(\boldsymbol{x})\hat{\pi}^\dagger(\boldsymbol{x}) - \frac{m^2c^2}{2\hbar^2}\hat{\phi}^\dagger(\boldsymbol{x})\hat{\phi}(\boldsymbol{x})\right), \tag{89}$$

resulting in

$$i\hbar\frac{d|\psi(t)\rangle}{dt} = \left(\hat{H}_0 + G\hat{\tilde{H}}_{int}\right)|\psi(t)\rangle. \tag{90}$$

We can expand $|\psi(t)\rangle$ to first order in G: $|\psi(t)\rangle =: |\psi(t)\rangle_0 + G|\psi(t)\rangle_1$, where in our case $|\psi(t)\rangle$ is given by (12), such that we are expanding the coefficients $\alpha_{ij}(t)$ in G. Since it depends on $|\psi(t)\rangle$, the potential $\tilde{\Phi}[\psi(t)](\boldsymbol{x})$ can also be expanded to first order in G:

$$\tilde{\Phi}[\psi(t)](\boldsymbol{x}) = \tilde{\Phi}_0[\psi(t)](\boldsymbol{x}) + G\tilde{\Phi}_1[\psi(t)](\boldsymbol{x}) + \cdots, \tag{91}$$

where:

$$\tilde{\Phi}_0[\psi(t)](\boldsymbol{x}) := \int d^3\boldsymbol{x}'\,\frac{{}_0\langle\psi(t)|\hat{T}_{00}(\boldsymbol{x}')|\psi(t)\rangle_0}{|\boldsymbol{x}-\boldsymbol{x}'|}, \tag{92}$$

$$\tilde{\Phi}_1[\psi(t)](\boldsymbol{x}) := \int d^3\boldsymbol{x}'\,\frac{2\mathrm{Re}\left({}_1\langle\psi(t)|\hat{T}_{00}(\boldsymbol{x}')|\psi(t)\rangle_0\right)}{|\boldsymbol{x}-\boldsymbol{x}'|}. \tag{93}$$

To first order in G, we then have:

$$i\hbar\frac{d|\psi(t)\rangle}{dt} = \left(\hat{H}_0 + G\hat{\tilde{H}}^0_{int}\right)|\psi(t)\rangle, \tag{94}$$

where

$$\hat{\tilde{H}}^0_{int} = \frac{4}{c^2}\int d^3\boldsymbol{x}\tilde{\Phi}_0[\psi(t)](\boldsymbol{x})\left(\hat{\pi}(\boldsymbol{x})\hat{\pi}^\dagger(\boldsymbol{x}) - \frac{m^2c^2}{2\hbar^2}\hat{\phi}^\dagger(\boldsymbol{x})\hat{\phi}(\boldsymbol{x})\right), \tag{95}$$

and

$$i\hbar\frac{d|\psi(t)\rangle_0}{dt} = \hat{H}_0|\psi(t)\rangle_0. \tag{96}$$

As discussed above, $\hat{H}_0$ can be shown to just act a global phase and as such we can take $|\psi\rangle_0 = |\psi(0)\rangle$ and thus $G\tilde{\Phi}_0[\psi(t)](\boldsymbol{x}) = \Phi(\boldsymbol{x})$ with $\Phi(\boldsymbol{x})$ given by Equation (52) and thus independent of time or the evolution of the quantum state of matter. Then to first order we simply have:

$$i\hbar\frac{d|\psi(t)\rangle}{dt} = \left(\hat{H}_0 + \hat{H}_{int}\right)|\psi(t)\rangle, \tag{97}$$

with $\hat{H}_{int}$ and $\Phi(\boldsymbol{x})$ given by (41) and (52), and we only keep solutions up to first order in G. We, therefore, to first order have the *linear* equation of quantum field theory in curved spacetime, but with $\Phi(\boldsymbol{x})$ given by (52).

We can continue the above procedure up to fourth order in G where the entanglement effect occurs, noting that, as shown above, the lower orders only contribute to a relative phase between the left and right states, such that $\Phi[\psi(t)](\boldsymbol{x})$ remains $\Phi(\boldsymbol{x})$ to the appropriate order. As such, the state to fourth order still obeys the linear Schrödinger equation (97), which can be solved through the Dyson series as above as long as we only consider the result up to, in general, the fourth order.

2.3 Discussion

It could be thought that since there are no virtual graviton propagators, it is not the gravitational interaction that is creating the above entanglement. To see that the gravitational interaction is indeed responsible for entanglement, we can consider what happens if the interaction Hamiltonian $\hat{H}_{int}$ for gravity is turned off, i.e. $\hat{H}_{int} = 0$. In this case, we just have the free evolution in (39), which just contributes a global phase as detailed in the quantum gravity section above and below Equation (24). It is then only once the interaction Hamiltonian for classical gravity $\hat{H}_{int}$ in (39) is turned on that we get entanglement.

We can also see this from the Feynman diagram for the process, Figure 2e in the main text: with $\hat{H}_{int}$ turned off, there will be no vertices, and the only way to create entanglement would be if a real particle from $1i$ freely propagates (diffuses) to $2j$. However, we have assumed fixed position-like modes for the experiment - see Equation (9) and (24) - such that this contribution is vanishingly small, leaving approximately just a global phase as discussed around (24). With the interaction turned on, vertices can be reintroduced which provide additional momentum to flow from gravity (as understood from the Fourier transform of $\Phi(\boldsymbol{x})$). If we just kept one vertex, say where $1i$ connects to the potential, then we have diagram 1b, which could still be thought of as involving diffusion of a real particle from $1i$ to $2j$. However, this diagram evaluates to zero as discussed around Equation (38). One way to see this is that the process is forbidden from energy-momentum conservation: while additional momentum flows into the vertex from gravity, it is not then possible to respect the energy-momentum relation for a real particle propagating out of (as well as into) the vertex. This illustrates that the particle must be off-shell, it must be virtual, and, therefore, there must be another vertex at $2j$. That is, only the diagram Figure 2e with interaction vertices and virtual (not real) particles propagating between the objects can create entanglement, further illustrating that $\hat{H}_{int}$ is essential to the process - without it, there would be no observable entanglement. We see then that the classical gravity interaction is responsible for creating the observed entanglement.That the size of entanglement is proportional to G, Equation (84), also illustrates that gravity is required for the entanglement process.

Note that, in the non-relativistic gravity limit, the interaction Hamiltonian for classical gravity Equation (41) is not, by itself, a spatial entangling operator for the matter field. That is, since all the operators act on the same spatial position, and there are no spatial derivatives, the operator cannot by itself entangle different spatial regions of the field. Instead, it is the free Hamiltonian $\hat{H}_0$ that contains the spatial derivatives required, in principle, for spatial entanglement - although, as discussed above, $\hat{H}_0$ by itself is also not enough to create observable entanglement due to the conditions set by the experiment. Therefore, we need *both* $\hat{H}_{int}$ and $\hat{H}_0$ to generate the observable entanglement. This can be seen from the fact that in the interaction picture $\hat{H}_{int}$ picks up time dependence through $\hat{H}_0$ resulting in the Dyson series, and equivalently from the Feynman diagrams: $\hat{H}_{int}$ provides the vertices and $\hat{H}_0$ the free propagation of the virtual particles in Figure 2e, with both effects required to generate the entanglement process as discussed above. This is also the case for the quantum gravity effect where the spatial matter field derivatives in the full relativistic interaction Hamiltonian (Equation 2 in the main text) play no role in generating entanglement due to the assumed non-relativistic in and out matter states (23) - see the discussion above (25). This can be seen more clearly when taking the same simplifying non-relativistic limit assumed for the classical gravity interaction - the corresponding interaction Hamiltonian in this case for quantum gravity is (41) but with a hat added to $h(\boldsymbol{x})$:

$$\hat{H}_{int} = -\int d^3\boldsymbol{x}\, \hat{h}(\boldsymbol{x})\Big(\hat{\pi}(\boldsymbol{x})\hat{\pi}^\dagger(\boldsymbol{x}) - \frac{m^2c^2}{2\hbar^2}\hat{\phi}^\dagger(\boldsymbol{x})\hat{\phi}(\boldsymbol{x})\Big), \tag{98}$$

with $\overline{\hat{h}(x)\hat{h}(y)}$ derivable from (18) [7]. This is also not a spatially entangling operator and, as with the classical gravity interaction, in order to generate the quantum-gravity entanglement, *both* $\hat{H}_{int}$ and $\hat{H}_0$ (in this case $\hat{H}_0^G$) are required. As with classical gravity, we can see this from the relevant Feynman diagram (Figure 1a in the main text) where $\hat{H}_{int}$ provides the vertices and $\hat{H}_0$ provides the free propagation of the virtual gravitons [7].

The previous proofs on entanglement only being generated by quantum or non-local theories of gravity [5, 6, 26–28] are violated here due to the theorems taking a restrictive view on what the

classical gravity interaction consists of. This restriction is that quantum gravity should only involve virtual graviton propagators, but we know from QED that interactions at the field theory level also involve virtual matter propagators, which also occur in classical gravity as we have demonstrated.

The question of whether entanglement can evidence quantum gravity then fundamentally becomes a phenomenological one: in principle it depends on the parameters of the particular experiment being proposed, as discussed in the main text. This settles a long-standing debate [23] in the quantum information and quantum gravity communities on whether we need "theory-independent" proofs that quantum gravity and not realistic (local) theories of classical gravity can create entanglement, or whether it is enough to just consider entanglement as *strong evidence* of quantum gravity in a particular experiment. We have demonstrated that only the latter view is possible.

2.4 Virtual matter in quantum gravity

Although we have considered the above virtual matter process from a classical gravity perspective, virtual matter processes will also exist in a quantum theory of gravity. In this case, the gravitational potential as well as the virtual matter will be in a quantum superposition. That is, Equation (70) for $\beta_{ij}^{(4)}$ becomes:

$$\kappa_{ij}^{(4)} := \frac{m^6 t^2 N^2}{4\pi^2\hbar^6}\left(i\int d^3\boldsymbol{x}\int d^3\boldsymbol{y}\,\frac{\Phi_{ij}(\boldsymbol{x})\,\Phi_{ij}(\boldsymbol{y})\,\theta_{1i}(\boldsymbol{x})\,\theta_{2j}(\boldsymbol{y})}{|\boldsymbol{x}-\boldsymbol{y}|}\right)^2, \tag{99}$$

where:

$$\Phi_{ij}(\boldsymbol{x}) := \Phi_{1i}(\boldsymbol{x}) + \Phi_{2j}(\boldsymbol{x}), \tag{100}$$

with:

$$\Phi_{\kappa i}(\boldsymbol{x}) := -GM\left[\left(\frac{3}{2R} - \frac{|\boldsymbol{x}-\boldsymbol{X}_{\kappa i}|^2}{2R^3}\right)\theta(R - |\boldsymbol{x}-\boldsymbol{X}_{\kappa i}|) + \frac{\theta(|\boldsymbol{x}-\boldsymbol{X}_{\kappa i}| - R)}{|\boldsymbol{x}-\boldsymbol{X}_{\kappa i}|}\right]. \tag{101}$$

This is straightforwardly derived from the quantum gravity Hamiltonian in the full Newtonian regime - Equation (42) with a hat added to $\Phi(x)$ to indicate that it can be superposed:

$$\hat{H}_I = \frac{4}{c^2}\int d^3\boldsymbol{x}\,\hat{\Phi}(\boldsymbol{x})\Big(\hat{\pi}(x)\hat{\pi}^\dagger(x) - \frac{m^2c^2}{2\hbar^2}\hat{\phi}^\dagger(x)\hat{\phi}(x)\Big), \tag{102}$$

and with $\hat{\Phi}(\boldsymbol{x})$ now written as:

$$\hat{\Phi}(\boldsymbol{x}) = -\frac{G}{c^2}\int d^3\boldsymbol{y}\,\frac{\hat{T}_{00}(\boldsymbol{y})}{|\boldsymbol{x}-\boldsymbol{y}|}. \tag{103}$$

Using (23), we then have ${}_{1i}\langle N|\hat{\Phi}(\boldsymbol{x})|N\rangle_{1i} = \Phi_{1i}(\boldsymbol{x})\,{}_{1i}\langle N|N\rangle_{1i} = \Phi_{1i}(\boldsymbol{x})$ and ${}_{1i}\langle N|\,{}_{2j}\langle N|\hat{\Phi}(\boldsymbol{x})|N\rangle_{1i}|N\rangle_{2j} = \Phi_{1i}(\boldsymbol{x}) + \Phi_{2j}(\boldsymbol{x})$ in the approximation $R \gg \hbar/(mc)$ used to describe the experiment (and since the spheres do not overlap). That is, $\hat{\Phi}(\boldsymbol{x})$ directly acts on the Hilbert space of matter [23].

The relevant process then derives at fourth order as in the classical gravity case, with the amplitude $\kappa_{ij}^{(4)}$ deriving from:

$$\kappa_{ij}^{(4)} = \frac{1}{16\,\hbar^4c^4}\int_t d^4x\,d^4y\,d^4z\,d^4w\,\times$$

$${}_{1i}\langle N|\,{}_{2j}\langle N|\hat{\Phi}(\boldsymbol{w})\hat{\Phi}(\boldsymbol{z})\hat{\Phi}(\boldsymbol{y})\hat{\Phi}(\boldsymbol{x})\hat{\mathcal{T}}_{\mu\nu}[\hat{\phi}^\dagger(w)\hat{\phi}(w)]\hat{\mathcal{T}}_{\rho\sigma}[\hat{\phi}^\dagger(z)\hat{\phi}(z)]\hat{\mathcal{T}}_{\gamma\delta}[\hat{\phi}^\dagger(y)\hat{\phi}(y)]\hat{\mathcal{T}}_{\kappa\lambda}[\hat{\phi}^\dagger(x)\hat{\phi}(x)]|N\rangle_{1i}\,|N\rangle_{2j},$$

where one could also act symbolic Wick contractions between $\hat{\Phi}(\boldsymbol{x})$ and the matter states, noting that $\hat{\Phi}(\boldsymbol{x})$ leaves the states intact. Alternatively, the process can also be derived at higher order from the full relativistic Hamiltonian (5), where there are *virtual gravitons* as well as virtual matter mediating between the masses. That is, although we can have only virtual matter exchange in classical gravity, in quantum gravity this exchange is always accompanied by graviton exchange in the experiment, such that you cannot strictly separate the two effects and there is always graviton exchange.

With (100) inserted into (99), the spatial integrals can be solved using the same method as in Section 2.1. In the approximation that $R \ll \Delta x$ and $R \ll d_{ij}$, which were also assumed in Section 2.1, we find:

$$\kappa_{ij}^{(4)} = \left(\frac{24}{25} \frac{iG^2 m^2 M^3 Rt}{\hbar^3 d_{ij}} \right)^2 , \tag{104}$$

which has the same form as the semi-classical case (84) except for a slightly larger numerical factor due to the 1/2 coming from the average of the potentials (52) compared to (100). The higher factor can also be considered as due to there being a superposition of gravitons *and* virtual matter, with both contributing to the entanglement in quantum gravity, whereas it is only the latter in a classical theory of gravity. With $d_{RL} \ll \Delta x$, the amplitude $\kappa_{RL}^{(4)}$ dominates over all others, with:

$$\kappa_{RL}^{(4)} = \left(\frac{24}{25} \frac{iG^2 m^2 M^3 Rt}{\hbar^3 d_{RL}} \right)^2 . \tag{105}$$

3 Non-local, "classical" theories of gravity

As mentioned in the main text, there have been several works that consider whether the theorems for how entanglement evidences quantum gravity are violated [13–16, 29–33]. Often inspired by discussions at the Chapel Hill conference where Feynman first introduced his experiment [34], these works have focused on whether classical gravity could act through non-local operations, violating the LO part of LOCC, which was also discussed in the original works on entanglement evidencing quantum gravity [5, 6, 35]. For example, there are arguments that, since the experiments operate in the low-energy regime of quantum gravity, gravity in the real-world must or could act as Newtonian gravity [14, 33, 34, 36] (despite experiments evidencing the relativistic nature of gravity [23]), with Newtonian gravity non-local; that general relativity is non-local since there are gauges in which it can appear this way [13, 14, 31, 34]; that we cannot be sure that there is a gravitational field such that gravity could act as a non-local constraint or a 'quantum-controlled' field similar to the absorber theory of electromagnetism by Feynman and Wheeler (there are no independent gravitational degrees of freedom) [15, 16, 37]; that classical gravity could act through de Broglie–Bohm theory [29, 30], which is inherently non-local; that classical gravity can be associated with a continuous monitoring of matter that is non-local [38, 39]; or that there is no true meaning to the concept of subsystems and thus locality [31, 32].

In fact, it is also possible to argue that these non-local operations should still be considered as (non-local) *quantum* theories of gravity rather than "classical" theories [33, 34]. For example, for entanglement to be generated with Newtonian gravity, the Newtonian gravitational force must itself go into a quantum superposition, which is in contrast to a Schrödinger-Newton or semi-classical theory of gravity, where the force or gravitational potential can never be in a quantum superposition since it is sourced by the quantum expectation of quantum matter (see above). That is, in the above works, the gravitational potential or gravitational field, implicitly, or explicitly, acts as a quantum operator; an approach we do not consider here. For example, in quantum-controlled gravity, the gravitational field or Newtonian potential is assumed explicitly different in each superposition branch of the experiment [15], whereas the gravitational field remains the same in each branch in the process we consider here. This can be seen, for example, in Equation 9 in the main text where $\Phi(\boldsymbol{x})$ is the same in each branch. Instead it is the function $\theta_{1i}(\boldsymbol{x})\theta_{2j}(\boldsymbol{y})$ coming from the matter sector that is different in each superposition branch, such that $\theta_{\kappa i}(\boldsymbol{x})$ could be considered to act as an implicit quantum operator. However, the function $\theta_{\kappa i}(\boldsymbol{x})$ being different in each branch is not enough to create entanglement - see e.g. (45) - we also need the $|\boldsymbol{x} - \boldsymbol{y}|$ term from the virtual matter propagator connecting the two

$\theta_{\kappa i}(\boldsymbol{x})$ functions (matter sectors). That is, what is generating entanglement in the process we consider is the local exchange of virtual matter, a process not considered previously for the experiments.

The possibility perhaps remains (although it has not been developed) for an unusual theory of gravity which acts non-locally but for which gravity itself is classical (the gravitational potential or force cannot be in a quantum superposition). At a fundamental level there seems to be no way to tell through entanglement whether gravity is local and quantum or just non-local. However, non-locality (action-at-a-distance) is usually ruled out on purely physical grounds [23], as discussed in the main text.

4 Stochastic classical gravity

As argued in the main text, the existence of a classical gravitational interaction implies, when quantum matter is treated using QFT, the possibility of having a matter propagator, which can generate entanglement regardless of the specific form of the classical gravity model. The virtual matter process considered in Section 5.2 in the main text is thus expected to exist in any fundamental classical gravity theory. However, the size of the effect will depend on how exactly gravity is sourced by quantum matter. In the main text and Section 2, we considered the most considered approach to sourcing gravity from matter in a fundamental theory of classical gravity. This is where gravity is sourced from the quantum expectation of the stress-energy operator of matter: $G_{\mu\nu} = \kappa\langle\hat{T}_{\mu\nu}\rangle$, where $\kappa = 8\pi G/c^4$, $G_{\mu\nu}$ is the Einstein tensor, and the average depends on the particular theory chosen. For example, a straightforward Everettian interpretation where the expectation is over the global wavefunction is ruled out through experiment [40], but other theories, such as that with Copenhagen-like collapse, or with the average over the 'local' matter states, have not been tested - see Section 5 for more detail.

In this section, we consider theories of classical gravity that involve fundamental stochacisty. For example, classical gravity could be sourced from stochastic fluctuations around the the quantum expectation of the stress-energy operator of matter [38, 41]: $G_{\mu\nu} = \kappa\langle\hat{T}_{\mu\nu}\rangle + \delta T_{\mu\nu}$, where $\delta T_{\mu\nu}$ is a stochastic quantity. In the non-relativistic limit this becomes:

$$\Phi(\boldsymbol{x}) = -\frac{G}{c^2}\int d^3\boldsymbol{x}'\,\frac{\langle\hat{T}_{00}(\boldsymbol{x}')\rangle + \delta T_{00}(\boldsymbol{x}')}{|\boldsymbol{x}-\boldsymbol{x}'|}. \tag{106}$$

Theories based on this equation have been developed, with T_{00} formally deriving from a continuous measurement process [38]. A relativistic theory of stochastic gravity also reproduces this result in the Newtonian limit [42, 43].

We now consider the dynamics of these theories. Just before taking the full Newtonian limit of the relativistic theory of stochastic gravity [42], the dynamics obey the following coupled equations when averaging over classical noise [43, 44]:

$$\frac{d\Phi(t,\boldsymbol{x})}{dt} = -\frac{1}{12}\partial_i n^i \tag{107}$$

$$\frac{d\pi(t,\boldsymbol{x})}{dt} = \frac{\nabla^2\Phi(t,\boldsymbol{x})}{4\pi G} - \langle\hat{m}(\boldsymbol{x})\rangle \tag{108}$$

$$\hbar\frac{d\hat{\rho}(t)}{dt} = -i[\hat{H}_0 + \hat{H}_{int}] + \frac{1}{2}\int d^3\boldsymbol{x}d^3\boldsymbol{y}D(\Phi,\boldsymbol{x},\boldsymbol{y})[\hat{m}(\boldsymbol{x}),[\hat{\rho}(t),\hat{m}(\boldsymbol{y})]], \tag{109}$$

where $\hat{\rho}(t)$ is the density operator for matter, $\hat{m}(\boldsymbol{x})$ is the mass density operator, π is the conjugate momentum of the dynamical classical field Φ, n^i is the shift vector of the ADM decomposition [43], $\hat{H}_0$ describes the free evolution, $D(\Phi,\boldsymbol{x},\boldsymbol{y})$ is a positive semi-definite kernel, and $\hat{H}_{int}$ is the non-relativistic version of Equation 4 in the main text:

$$\hat{H}_{int} = \int d^3\boldsymbol{x}\,\hat{m}(\boldsymbol{x})\Phi(\boldsymbol{x}). \tag{110}$$

The above coupled equations take the form of traditional semi-classical gravity in the non-relativistic regime (see Section 2) but with a decoherence term.

For the full Newtonian limit, one must apply the constraint $\pi \approx 0$ [42]. This results in the same dynamics for the potential and density operator as those derived using a formal continuos measurement process [38]. However, there is much freedom in these theories. Taking the perspective of a formal continuous measurement process, this freedom comes from a free choice in the spatial resolution of a single detector and the correlations of the outputs of the detectors [38]. However, by applying a principle of minimal decoherence [45], the latter freedom can be fixed, and the dynamical equation for $\hat{\rho}(t)$, after averaging over noise, becomes [45]:

$$\hbar \frac{d\hat{\rho}(t)}{dt} = -i \left[\hat{H}_0 + \frac{1}{2} \int d^3\boldsymbol{x}\, \hat{\Phi}(\boldsymbol{x}) \hat{m}(\boldsymbol{x}), \hat{\rho}(t) \right] + \frac{1}{2} \int d^3\boldsymbol{x} \left[\hat{\Phi}(\boldsymbol{x}), [\hat{m}(\boldsymbol{x}), \hat{\rho}(t)] \right], \tag{111}$$

with $\hat{\Phi}(\boldsymbol{x}) := -G \int d^3\boldsymbol{y}\, \hat{m}(\boldsymbol{y})/|\boldsymbol{x}-\boldsymbol{y}|$.

This is the Diósi-Penrose model [41, 46, 47] but with a Newtonian quantum gravity unitary term - the first term on the right-hand side of (111). Remarkably, although the gravitational potential is considered classical, see (106), mathematically the evolution of the density operator is equivalent to there being (Newtonian) quantum gravity (where the potential is effectively operator-valued, $\hat{\Phi}(\boldsymbol{x})$) but with sufficient decoherence to prevent quantum communication through the gravitational potential. There is still freedom in this theory due to the need to regularize the theory in order to keep it finite (for example, due to the infinite potential of point-like particles), which can be formally interpreted as describing a quantum system subjected to a continuous monitoring of its (smeared) mass density [38]. It is possible that this can lead to non-locality sufficient to create entanglement - for example, if the mass density is smeared as a Gaussian and over a distance greater than that between the objects such that the interpretation of subsystems becomes challenging [39]. To avoid this, we instead use smeared density functions with the step functions $\theta_{\kappa i}(\boldsymbol{x})$, as in the main text and Sections 1 and 2, such that there is no overlap in the density functions, avoiding any potential non-local effects.

We now apply the methodologies of Sections 1 and 2 to these theories in order to estimate the size of the entanglement effect through the virtual matter process considered in the main text. For this, we first upgrade (111) to relativistic complex scalar matter fields. This results in replacing $\hat{m}(\boldsymbol{x})$ with $\hat{\Pi}(\boldsymbol{x}) = \frac{4}{c^2}\left(\hat{\pi}(\boldsymbol{x})\hat{\pi}^\dagger(\boldsymbol{x}) - \frac{m^2c^2}{2\hbar^2}\hat{\phi}^\dagger(\boldsymbol{x})\hat{\phi}(\boldsymbol{x})\right) := \hat{\mathcal{T}}[\hat{\phi}^\dagger(\boldsymbol{x})\hat{\phi}(\boldsymbol{x})]$ (see Section 2), such that we have:

$$\hbar \frac{d\hat{\rho}(t)}{dt} = -i \left[\hat{H}_0 + \frac{1}{2} \int d^3\boldsymbol{x}\, \hat{\Phi}(\boldsymbol{x}) \hat{\Pi}(\boldsymbol{x}), \hat{\rho}(t) \right] + \frac{1}{2} \int d^3\boldsymbol{x} \left[\hat{\Phi}(\boldsymbol{x}), [\hat{\Pi}(\boldsymbol{x}), \hat{\rho}(t)] \right]. \tag{112}$$

Taking the non-relativistic limit $\hat{\phi}(\boldsymbol{x}) = \frac{\hbar}{\sqrt{2m}}\hat{\psi}(\boldsymbol{x})e^{-imcx^0/\hbar}$, where $\hat{\psi}(\boldsymbol{x})$ is a non-relativistic quantum field, results in (111) with $\hat{m}(\boldsymbol{x}) = m\,\hat{\psi}^\dagger(\boldsymbol{x})\hat{\psi}(\boldsymbol{x})$. We next switch to the interaction picture: we insert $\hat{\rho}(t) = \hat{U}_0\hat{\rho}_I(t)\hat{U}_0^\dagger$, where $\hat{U}_0 = e^{-i\hat{H}_0 t/\hbar}$, resulting in the removal of the free dynamics:

$$\begin{aligned} \frac{d\hat{\rho}_I(t)}{dt} &= -\frac{1}{2\hbar} i \int d^3\boldsymbol{x} \left[\hat{\Phi}(x)\hat{\Pi}(x), \hat{\rho}_I(t) \right] + \frac{1}{2\hbar} \int d^3\boldsymbol{x} \left[\hat{\Phi}(x), [\hat{\Pi}(x), \hat{\rho}_I(t)] \right] && (113)\\ &:= \left(\hat{\mathcal{L}}_U + \hat{\mathcal{L}}_D \right) \hat{\rho}_I(t) && (114)\\ &:= \hat{\mathcal{L}}\hat{\rho}_I(t) && (115) \end{aligned}$$

where $\hat{\Pi}(x)$ and $\hat{\Phi}(x)$ are the interaction picture versions of $\hat{\Pi}(\boldsymbol{x})$ and $\hat{\Phi}(\boldsymbol{x})$, and $\hat{\mathcal{L}}\hat{\rho} := \left(\hat{\mathcal{L}}_U + \hat{\mathcal{L}}_D\right)\hat{\rho}$, with $\hat{\mathcal{L}}_U\hat{\rho}$ and $\hat{\mathcal{L}}_D\hat{\rho}$ the superoperators:

$$\hat{\mathcal{L}}_U\hat{\rho} := -\frac{1}{2\hbar} i \int d^3\boldsymbol{x} \left[\hat{\Phi}(x)\hat{\Pi}(x), \hat{\rho} \right], \tag{116}$$

$$\hat{\mathcal{L}}_D\hat{\rho} := \frac{1}{2\hbar} \int d^3\boldsymbol{x} \left[\hat{\Phi}(x), [\hat{\Pi}(x), \hat{\rho}] \right]. \tag{117}$$

The solution to (115) can be formally written as:

$$\hat{\rho}_I(t) = \hat{T} e^{\int_0^t d\tau\, \hat{\mathcal{L}}(\tau)} \hat{\rho}(0) = \hat{T} e^{\int_0^t d\tau\, (\hat{\mathcal{L}}_U(\tau) + \hat{\mathcal{L}}_D(\tau))} \hat{\rho}(0) \tag{118}$$

$$= \hat{T}(1 + \int_0^t d\tau\, (\hat{\mathcal{L}}_U(\tau) + \hat{\mathcal{L}}_D(\tau)) \tag{119}$$
$$+ \frac{1}{2!} \int_0^t d\tau_1\, d\tau_2\, (\hat{\mathcal{L}}_U(\tau_1) + \hat{\mathcal{L}}_D(\tau_1))(\hat{\mathcal{L}}_U(\tau_2) + \hat{\mathcal{L}}_D(\tau_2))$$
$$+ \dots)\hat{\rho}(0).$$

The Schrödinger picture density operator is then obtained from $\hat{\rho}(t) = \hat{U}_0 \hat{\rho}_I(t) \hat{U}_0^\dagger$. Applying this to Feynman's experiment, the initial and final (before the reverse Stern-Gerlach) density operators will be the density operator versions of (8) and (12). Ignoring the spin states, the density operator can then be written as a 4×4 matrix, with the rows and columns labelled by $\{|N\rangle_{1L}, |N\rangle_{1R}, |N\rangle_{2L}, |N\rangle_{2R}\}$ and $\{{}_{1L}\langle N|, {}_{1R}\langle N|, {}_{2L}\langle N|, {}_{2R}\langle N|\}$ respectively. Given the final density operator $\hat{\rho}(t)$, we can obtain the different entries $\rho_{ij,kl}$ of the matrix by $\rho_{ij,kl}(t) = {}_{1i}\langle N|\, {}_{2j}\langle N|\, \hat{\rho}(t)\, |N\rangle_{1k} |N\rangle_{2l}$. As discussed in Section 1, $\hat{U}_0$ only acts a global phase, and thus we only need to consider this for the interaction picture density operator: $\rho_{ij,kl}(t) = {}_{1i}\langle N|\, {}_{2j}\langle N|\, \hat{\rho}_I(t)\, |N\rangle_{1k} |N\rangle_{2l}$.

To begin with we consider the first order term in (119). To determine the density matrix, we then need to calculate the contractions:

$${}_{1i}\langle N|\, {}_{2j}\langle N| \int_0^t d\tau\, c\hbar\, \hat{\mathcal{L}}_U(\tau) \hat{\rho}(0)\, |N\rangle_{1k} |N\rangle_{2l} \tag{120}$$

$$= \int_\tau d^4x\, {}_{1i}\langle N|\, {}_{2j}\langle N|\, \hat{\Phi}(x)\hat{\Pi}(x)\hat{\rho}(0)\, |N\rangle_{1k}|N\rangle_{2l} - \int_\tau d^4x\, {}_{1i}\langle N|\, {}_{2j}\langle N|\, \hat{\rho}(0)\hat{\Phi}(x)\hat{\Pi}(x)|N\rangle_{1k}|N\rangle_{2l} \tag{121}$$

$$= \int_\tau d^4x\, {}_{1i}\langle N|\, {}_{2j}\langle N|\, \hat{\Phi}(x)\hat{\Pi}(x)\, |N\rangle_{1i}|N\rangle_{2j} - \int_\tau d^4x\, {}_{1k}\langle N|\, {}_{2l}\langle N|\, \hat{\Phi}(x)\hat{\Pi}(x)|N\rangle_{1k}|N\rangle_{2l} \tag{122}$$

and

$${}_{1i}\langle N|\, {}_{2j}\langle N| \int_0^t d\tau\, c\hbar\, \hat{\mathcal{L}}_D(\tau) \hat{\rho}(0)\, |N\rangle_{1k} |N\rangle_{2l} \tag{123}$$

$$= \int_\tau d^4x\, {}_{1i}\langle N|\, {}_{2j}\langle N|\, \hat{\Phi}(x)\hat{\Pi}(x)\, |N\rangle_{1i}|N\rangle_{2j} + \int_\tau d^4x\, {}_{1k}\langle N|\, {}_{2l}\langle N|\, \hat{\Pi}(x)\hat{\Phi}(x)|N\rangle_{1k}|N\rangle_{2l} \tag{124}$$

$$- \int_\tau d^4x\, {}_{1k}\langle N|\, {}_{2l}\langle N|\, \hat{\Pi}(x)|N\rangle_{1k}|N\rangle_{2l} \times {}_{1i}\langle N|\, {}_{2j}\langle N|\, \hat{\Phi}(x)|N\rangle_{1i}|N\rangle_{2j} \tag{125}$$

$$- \int_\tau d^4x\, {}_{1k}\langle N|\, {}_{2l}\langle N|\, \hat{\Phi}(x)|N\rangle_{1k}|N\rangle_{2l} \times {}_{1i}\langle N|\, {}_{2j}\langle N|\, \hat{\Pi}(x)|N\rangle_{1i}|N\rangle_{2j}, \tag{126}$$

where we have used the orthonormality of the states in determining what components of $\hat{\rho}(0)$ contribute a non-zero result. We are now able to apply the contractions developed in Sections 1 and 2. For example,

$$\int_\tau d^4x\, {}_{1i}\langle N|\, {}_{2j}\langle N|\, \hat{\Phi}(x)\hat{\Pi}(x)\, |N\rangle_{1i}|N\rangle_{2j} \tag{127}$$

$$= \int_t d^4x\, (\Phi_{1i}(\boldsymbol{x}) + \Phi_{2j}(\boldsymbol{x}))\, {}_{1i}\langle N|\, {}_{2j}\langle N|\hat{\mathcal{T}}[\hat{\phi}^\dagger(x)\hat{\phi}(x)]|N\rangle_{1i}\, |N\rangle_{2j} \tag{128}$$

$$+ \int_t d^4x\, (\Phi_{1i}(\boldsymbol{x}) + \Phi_{2j}(\boldsymbol{x}))\, {}_{1i}\langle N|\, {}_{2j}\langle N|\hat{\mathcal{T}}[\hat{\phi}^\dagger(x)\hat{\phi}(x)]|N\rangle_{1i}\, |N\rangle_{2j}. \tag{129}$$

Using the contractions defined in 1, we can then determine the evolution of the density matrix for the matter system. We find that, to first order in (119):

$$\rho(t) = \rho(0) + \frac{1}{4}\times \begin{pmatrix} 0, & i\Delta U_{LRLL} - E_{G2}, & i\Delta U_{RLLL} - E_{G1}, & i\Delta U_{RRLL} - E_{GT} + \Delta U_{LLLR} + \Delta U_{RRRL} \\ -i\Delta U_{LRLL} - E_{G2}, & 0, & i\Delta U_{RLLR} - E_{GT} + \Delta U_{LRLL} + \Delta U_{RLLL}, & i\Delta U_{RRLR} - E_{G1} \\ -i\Delta U_{RLLL} - E_{G1}, & -i\Delta U_{RLLR} - (E_{GT} + \Delta U_{LRLL} + \Delta U_{RLRR}), & 0, & i\Delta U_{RRRL} - E_{G2} \\ -i\Delta U_{RRLL} - E_{GT}\Delta U_{LLLR} + \Delta U_{RRRL}, & -i\Delta U_{RRLR} - E_{G1}, & -i\Delta U_{RRLL} - E_{G2}, & 0 \end{pmatrix} t, \tag{130}$$

where:

$$\Delta U_{ijkl} := U_{ij} - U_{kl} \tag{131}$$

$$U_{ij} := \frac{GM^2}{\hbar\, d_{ij}}, \tag{132}$$

$$E_{GT} := E_{G1} + E_{G2} \tag{133}$$

$$E_{G\kappa} := -\frac{M}{\hbar V}\int d^3\boldsymbol{x}\, \Phi_{\kappa L}(\boldsymbol{x})\left(\theta_{\kappa L}(\boldsymbol{x}) - \theta_{\kappa R}(\boldsymbol{x})\right), \tag{134}$$

with U_{ij} the gravitational interaction energy between the masses 1 and 2 in states i and j respectively, and $E_{G\kappa}$ is the gravitational self-energy of the difference between the mass distributions of the two states of the solid object κ. The latter, which is the same for both objects κ in our case since the objects are identical (and so we denote E_G), is the usual rate of collapse in the Diósi-Penrose model for a single object in a superposition and is given by [25]):

$$E_G = \begin{cases} \frac{6GM^2}{5R}\left(\frac{5}{3}\lambda^2 - \frac{5}{4}\lambda^3 + \frac{1}{6}\lambda^5\right) & \text{if } 0 \le \lambda \le 1, \\ \frac{6GM^2}{5R}\left(1 - \frac{5}{12\lambda}\right) & \text{if } \lambda \ge 1, \end{cases}, \tag{135}$$

where $\lambda = \Delta x/(2R)$. The density matrix (130) is in fact the first order of the matrix one would obtain by non-perturbatively solving the Diòsi-Penrose model (with the unitary Newtonian quantum gravity term) for a non-relativistic version of Feynman's experiment [25, 45]:[5]

$$\rho(t) = \frac{1}{4}\times \begin{pmatrix} 1, & e^{it\Delta U_{LRLL}}e^{-E_{G2}t}, & e^{it\Delta U_{RLLL}}e^{-E_{G1}t}, & e^{it\Delta U_{RRLL}}e^{-(E_{GT}+\Delta U_{LLLR}+\Delta U_{RRRL})t} \\ e^{-it\Delta U_{LRLL}}e^{-E_{G2}t/\hbar}, & 1, & e^{it\Delta U_{RLLR}}e^{-(E_{GT}+\Delta U_{LRLL}+\Delta U_{RLLL})t}, & e^{it\Delta U_{RRLR}}e^{-E_{G1}t} \\ e^{-it\Delta U_{RLLL}}e^{-E_{G1}t}, & e^{-it\Delta U_{RLLR}}e^{-(E_{GT}+\Delta U_{LRLL}+\Delta U_{RLRR})t}, & 1, & e^{it\Delta U_{RRRL}}e^{-E_{G2}t/\hbar} \\ e^{-it\Delta U_{RRLL}}e^{-(E_{GT}\Delta U_{LLLR}+\Delta U_{RRRL})t}, & e^{-it\Delta U_{RRLR}}e^{-E_{G1}t/\hbar}, & e^{-it\Delta U_{RRLL}}e^{-E_{G2}t/\hbar}, & 1 \end{pmatrix}. \tag{136}$$

This illustrates that the methodology developed in Sections 1 and 2 can be successfully applied to this class of classical gravity models.

With $d_{RL} \ll \Delta x$ and $\Delta x \gg R$, we can approximate (136) by:

$$\rho(t) = \frac{1}{4}\begin{pmatrix} 1 & e^{-E_G t} & e^{-E_G t}e^{iU_{RL}t} & e^{-(2E_G - U_{RL})t} \\ e^{-E_G t} & 1 & e^{-(2E_G+U_{RL})t}e^{iU_{RL}t} & e^{-E_G t} \\ e^{-E_G t}e^{-iU_{RL}t} & e^{-(2E_G+U_{RL})t}e^{-iU_{RL}t} & 1 & e^{-E_G t}e^{-iU_{RL}t} \\ e^{-(2E_G-U_{RL})t} & e^{-E_G t} & e^{-E_G t}e^{iU_{RL}t} & 1 \end{pmatrix},$$

with $E_G = 6GM^2/(5R)$. In each off-diagonal entry of the density matrix, we have a decoherence term coming through $\sigma_G := E_G t$. Since this is always greater than $\varphi_{RL} = U_{RL}t$, which contributes a

[5] Assuming that at $t = 0$ we are able to start in a quantum superposition state.

quantum phase, no entanglement is generated here. That is, although we appear to have the quantum gravity-induced phase φ_{RL}, any entanglement it could generate gets cancelled by the decoherence process σ_G. As long as we keep to first order in the entanglement measure, the same applies to the matrix we directly calculated (130) - no entanglement is generated in this case.[6]

However, although σ_G is greater than φ_{RL}, it is possible for σ_G to be much smaller than $|\beta^{(4)}_{RL}|$ and its quantum-gravity version $|\kappa^{(4)}_{RL}|$ - (105). This is to be expected since, as described in the main text, $|\beta^{(4)}_{RL}|$ can be much greater than φ_{RL}, and σ_G is of the same form as φ_{RL} (same dependence on mass, Planck's constant etc.), and can be made close to φ_{RL}. For example, taking Ytterbium masses [5] with masses even as large as $M = 10\,\mathrm{mg}$ (and $d_{RL} \approx 10R$), $|\kappa^{(4)}_{RL}|$ is greater than σ_G for times larger than just $t \approx 10^{-19}\mathrm{s}$ (it is $\kappa^{(4)}_{RL} = 16 \times \beta^{(4)}_{RL}$ that is of relevance here rather than $\beta^{(4)}_{RL}$ since mathematically the density matrix evolves as if there is quantum gravity with decoherence, rather than semi-classical gravity with decoherence). Thus it is possible for the rate of entanglement generation from the virtual matter process to overcome the rate of decoherence σ_G. However, to determine if entanglement definitively occurs in this model, we need to see if there are any other decoherence processes that have a rate that is always greater than or equal to $|\kappa^{(4)}_{RL}|$. For this to occur, such a process must have the same dependence on experimental parameters as $|\kappa^{(4)}_{RL}|$, for example the mass m. Therefore, we need to consider processes involving virtual matter propagators.

The first candidate for such a decoherence process occurs at second order in the perturbation series (119) such that we need to consider terms of the form:

$$\hat{T}\frac{1}{2!}\,{}_{1i}\langle N|\,{}_{2j}\langle N|\int_0^t d\tau_1\, d\tau_2\,\Big[\hat{\mathcal{L}}_U(\tau_1)\hat{\mathcal{L}}_U(\tau_2)+\hat{\mathcal{L}}_U(\tau_1)\hat{\mathcal{L}}_D(\tau_2) + \hat{\mathcal{L}}_D(\tau_1)\hat{\mathcal{L}}_U(\tau_2)+\hat{\mathcal{L}}_D(\tau_1)\hat{\mathcal{L}}_D(\tau_2)\Big]\hat{\rho}(0)\,|N\rangle_{1k}|N\rangle_{2l}. \tag{137}$$

However, since we are only interested in contractions involving virtual matter propagators, we only need to consider those terms where $\hat{\rho}(0)$ is on the far right, far left, or where there is one or two $\hat{\Phi}$ terms on the far right or left. For example, considering the term ${}_{1i}\langle N|\,{}_{2j}\langle N|\int_0^t d\tau_1\, d\tau_2\,[\hat{\mathcal{L}}_U(\tau_1)\hat{\mathcal{L}}_U(\tau_2)]\hat{\rho}(0)\,|N\rangle_{1k}|N\rangle_{2l}$, with $\hat{\rho}(0)$ on the far right, we have the contractions (55) discussed in Section 2 but with $\Phi(\boldsymbol{x})$ replaced with $\hat{\Phi}(x)$:

$$\int_t d^4x\, d^4y\;{}_{1i}\langle N|\,{}_{2j}\langle N|\hat{\Phi}(y)\hat{\Phi}(x)\hat{\mathcal{T}}[\hat{\phi}^\dagger(y)\hat{\phi}(y)]\hat{\mathcal{T}}[\hat{\phi}^\dagger(x)\hat{\phi}(x)]|N\rangle_{1i}\,|N\rangle_{2j}$$
$$+\int_t d^4x\, d^4y\;{}_{1i}\langle N|\,{}_{2j}\langle N|\hat{\Phi}(y)\hat{\Phi}(x)\hat{\mathcal{T}}[\hat{\phi}^\dagger(y)\hat{\phi}(y)]\hat{\mathcal{T}}[\hat{\phi}^\dagger(x)\hat{\phi}(x)]|N\rangle_{1i}\,|N\rangle_{2j}, \tag{138}$$

whereas, with $\hat{\rho}(0)$ on the left, we have the same as above but with $1i$ replaced with $1k$. As discussed in Section 2, the corresponding contractions to the above - (55) - in the classical gravity models considered there, only contribute a relative phase and thus no entanglement. Even so, it is possible that they could contribute towards a decoherence process that can destroy any entanglement generated through the $\kappa^{(4)}_{ij}$ process. However, we show below that this is not the case.

The contractions in (138) involve those that look like the virtual matter equivalent to the E_G process above - the dependence on mass is the same as $\sqrt{\kappa^{(4)}_{ij}}$, but there are terms where we are integrating the potentials over their own source. For example:

$$\frac{1}{4\hbar^2c^2}\int_t d^4x\, d^4y\;{}_{1R}\langle N|\,{}_{2L}\langle N|\Phi_{1L}(\boldsymbol{y})\hat{\Phi}_{1L}(\boldsymbol{x})\hat{\mathcal{T}}[\hat{\phi}^\dagger(y)\hat{\phi}(y)]\hat{\mathcal{T}}[\hat{\phi}^\dagger(x)\hat{\phi}(x)]|N\rangle_{1R}\,|N\rangle_{2L} \tag{139}$$

$$= \frac{m^3Nt}{2\pi\hbar^3V}\int d^3\boldsymbol{x}d^3\boldsymbol{y}\frac{\Phi_{1L}(\boldsymbol{x})\Phi_{1L}(\boldsymbol{y})\theta_{1L}(\boldsymbol{x})\theta_{1L}(\boldsymbol{y})}{|\boldsymbol{x}-\boldsymbol{y}|}. \tag{140}$$

[6]For a discussion on how Tilloy-Diósi models of gravity can create entanglement (through non-locality), see [39].

For all terms in (137), for $\hat{\rho}(0)$ on the right, we get positive contributions from the commutations, such that there is an overall factor of $(1-i)^4 = -2i$ to the above process. In contrast, when $\hat{\rho}(0)$ is on the left, we get negative contributions with $\hat{\mathcal{L}}_U\hat{\mathcal{L}}_D$ and $\hat{\mathcal{L}}_D\hat{\mathcal{L}}_U$, giving an overall contribution of $2i$ for $\hat{\rho}(0)$ on the left. The terms from $\hat{\rho}(0)$ on the left and on the right then cancel. This leaves the terms with one and two $\hat{\Phi}$ on the left and right. Following the same analysis as above, most terms cancel, leaving contributions that combine to provide a factor of $-4i$ to the right-hand side of (140). This 'self-interaction' contraction thus does not contribute towards a decaying (decoherence) term for the $\rho_{RL,LL}$ entry, and we find that it does not contribute a decoherence term for any entry of the density matrix. Therefore, the analogue of the E_G process with virtual matter propagators does not generate a decoherence process.

As well as the 'self-interaction' type term, there will also be other terms from (138). Of these, the greatest involve integrals of the form:

$$\int d^3\boldsymbol{x} d^3\boldsymbol{y} \frac{\Phi_{1i}(\boldsymbol{x})\Phi_{2j}(\boldsymbol{y})\theta_{1i}(\boldsymbol{x})\theta_{1i}(\boldsymbol{y})}{|\boldsymbol{x}-\boldsymbol{y}|} \propto \frac{1}{d_{ij}}, \tag{141}$$

which have the same proportionality with respect to d_{ij} as $\sqrt{\kappa_{ij}^{(4)}}$. In the assumption that $d_{RL} \ll \Delta x$, we only need consider contractions involving $1R$ and $2L$. Although this process contributes towards the first order of an exponential decay for some of the density matrix entries, unlike E_G above, it does not enter all off-diagonal entries. This can be seen, for example, by considering any density matrix entry that does not contain both $1R$ and $2L$ - in this case there is no process that contributes a $1/d_{RL}$ scaling, and so any processes for this entry can be neglected. Overall, this process then does not create decoherence - it does not suppress entanglement generation.

Finally, we now consider the fourth order of the expansion (119). The processes at this order will clearly involve the entanglement process considered in Section 2 (in fact, they will involve the quantum gravity version - see Section 2.4). For a decoherence process to contribute the same dependence with experimental parameters as $\kappa_{ij}^{(4)}$, there needs to be two virtual matter propagators at this order. We have already seen that 'self-interaction' terms (140) at second order do not contribute a decoherence process, and thus these do not need to be considered at fourth order. This then leaves only those terms in (119) at fourth order where $\hat{\rho}(0)$ is on the far left or right, and when there are Φ, Φ^2, Φ^3 and Φ^4 on the right and left. With the assumption that $d_{RL} \ll \Delta x$, we also only need consider terms that will result in the expectations ${}_{1R}\langle N|\,{}_{2L}\langle N|\cdots|N\rangle_{1R}|N\rangle_{2L}$. Clearly, as at second order, any such decoherence process will not affect all off-diagonal entries. If, however, it contributed to the first order of a exponential decay in the entries of the density matrix where we have the $\beta_{RL}^{(4)}$ process generating entanglement in the classical gravity theories of Section 2 (and Section 2.4), then we might expect entanglement to never be generated in the theory considered in this section. This, however, is not the case. For example, considering ${}_{1L}\langle N|\,{}_{2L}\langle N|\hat{\rho}_I(t)|N\rangle_{1R}|N\rangle_{2L}$ at fourth order, and applying the arguments above, in the approximation $d_{RL} \ll \Delta x$, there is just the contribution $i\vartheta_{RL}$, which does not contribute to an exponential decay.

In summary, there is no entanglement-destroying processes besides the σ_G process up to fourth order where the entanglement generation occurs. This means that if $|\kappa_{RL}^{(4)}| \gg \sigma_G$, then entanglement occurs and with a rate that can be 16 times larger than for the classical gravity theories considered in Section 2 and the main text, since $\kappa_{RL}^{(4)} = 16 \times \beta_{RL}^{(4)}$. That entanglement in general occurs in these theories is not unexpected: the decoherence in (112) due to stochasticity is, mathematically, to prevent virtual gravitons from generating quantum communication (since gravity is classical and thus there cannot be quantum communication from gravitons), but there is no reason why there needs to be a decoherence process in these theories to, mathematically, prevent quantum communication from virtual matter since this is present even when gravity is classical (see the main text). As long then as the virtual matter process dominates over the (mathematical) graviton process, which is of course the regime of relevance to experiments seeking quantum gravity effects (see the main text), then the entanglement from virtual matter overcomes the decoherence preventing the, mathematical, virtual graviton exchange.

Similar to virtual graviton exchange in quantum gravity, the process we have considered here for generating entanglement can be viewed as coming from local, quantum communication. It is also possible, depending on the chosen stochastic classical gravity model, that there could be an additional non-local process generating entanglement, as argued in [38, 39]. This is a very different process to the one we have considered here since it is not through virtual matter exchange, is non-local and can be viewed as due to pre-existing quantum correlations between fictitious detectors that are continuously monitoring the matter distributions [38], as well as potential issues associated with modelling subsystems due to the spatial resolution of the detectors [38]. As argued in the main text, on physical grounds, such non-local processes would generally want to be avoided in a realistic model of nature [42].

5 Consistency of fundamentally classical gravity

The theoretical consistency of fundamentally classical gravity has been much debated. In particular, three different works [48], [40] and [49] have had a major impact. In Ref. [40], an experiment was performed that was designed to demonstrate that semi-classical gravity, where gravity is sourced by the expectation value of quantum matter, should be ruled out. However, this is only possible for the most straightforward approach of coupling the many-worlds interpretation of quantum theory to classical gravity [21] and, therefore, the experiment does not rule out semi-classical gravity in general. Ref. [48], argues that, in a fundamental theory of classical gravity, and in the Copenhagen interpretation of quantum mechanics [50], if a gravitational wave interacts with quantum matter then either the Heisenberg uncertainty relations must be violated, momentum conservation is violated or we must have superluminal signalling. Whether a violation of the Heisenberg uncertainty relations or momentum conservation should rule out a theory is up for debate, however, in any case, it has been demonstrated [51] that the argument for violation of the Heisenberg uncertainty relations (which is based on Heisenberg's controversial 'observer effect' interpretation of his relations [52, 53]) and momentum conservation in Ref. [48] is false, and therefore the arguments in Ref. [48] do not prove that fundamentally classical gravity leads to a violation in either the Heisenberg uncertainty relations, momentum conservation or the no-superluminal signalling principle of quantum mechanics. Furthermore, Ref. [51] showed that physically demonstrating any violation of the Heisenberg uncertainty relations, momentum conservation or no superluminal signalling as suggested in Ref. [48], would not be physically possible such that, even if there were a violation in principle, it would not show up in practice.

In Ref. [49], it was argued that non-linear modifications to quantum mechanics generally lead to superluminal signalling. Fundamental classical gravity theories where gravity is sourced by the expectation of matter (without stochastic fluctuations) are examples of non-linear modifications to quantum mechanics (see Section 2.2) - for example, see [54] for a discussion on how superluminal signalling could arise in this way in the Schrödinger–Newton equation. However, Ref. [55] demonstrated that superluminal signalling does not necessarily lead to a contradiction, and it is also possible to avoid any signalling by generalizing the sourcing of gravity such that the expectation value is taken over 'local' states of matter [20, 21, 56], where wavefunction collapse essentially becomes a real and relativistic process in the gravity sector [20–22, 56], or self-consistently generalizing the measurement and states of the theory [57]. It is also unclear if the superluminal signalling could be physically demonstrated [51].

In the main text, after deriving the general result Equation 9, showing that entanglement would generally occur in a classical theory of gravity, we specialized to semi-classical gravity where the Newtonian potential is sourced by the expectation of the matter states (see also Section 2). Using a perturbative analysis, we showed that entanglement occurs in this theory for Feynman's experiment. In Section 2.2, we demonstrated that in this perturbative regime, the theory is linear. Therefore, the entanglement result is not coming from a superluminal signalling process, which would question the locality and significance of the effect. Furthermore, as detailed in Section 5.1, the analysis is unchanged if we adopt the Newtonian potential as being sourced by 'local' matter states [20, 21] or where wavefunction collapse is a relativistic process [22] such that superluminal signalling can clearly not occur [20, 22]. This is because, in determining whether the quantum systems become entangled, we only need consider the time evolution of the quantum system (due to the classical gravity interaction

between the objects) after it is sent, for example, through the forward Stern-Gerlach devices and before the reverse devices, and no measurement processes occur during this period of Feynman's experiment. It is only at the end of the experiment that a measurement is performed, by which point the matter states are no longer in a superposition and entanglement can be transferred, for example, to the internal spin sector [5]. The experiment is also far from the regime of Ref. [48] where gravitational waves are scattered of masses and then precisely analysed (indeed, as argued in [51], no physical experiment may be in this regime).

Without updating the states or measurement process [20, 22, 57], we can see how a superluminal signalling could, in theory and in principle, be created using (a slightly modified version of) Feynman's experiment due to non-linearity. Consider that two matter objects are sent through the Stern-Gerlach devices so that they are in the superposition state as Equation 5 in the main text:

$$|\Psi\rangle = \frac{1}{2}\left(|N\rangle_{1L}|\uparrow\rangle_1 + |N\rangle_{1R}|\downarrow\rangle_1\right) \otimes \left(|N\rangle_{2L}|\uparrow\rangle_2 + |N\rangle_{2R}|\downarrow\rangle_2\right), \tag{142}$$

Immediately after this state is created, we assume that the two matter objects become entangled through, for example, an electromagnetic process (or potentially the classical gravity process considered in Section 2), placing them in the maximally entangled state:

$$\begin{aligned}|\Psi_-\rangle = \frac{1}{2}\Big(&|N\rangle_{1L}|\uparrow\rangle_1|N\rangle_{2L}|\uparrow\rangle_2 + |N\rangle_{1L}|\uparrow\rangle_1|N\rangle_{2R}|\downarrow\rangle_2 \\ &- |N\rangle_{1R}|\downarrow\rangle_1|N\rangle_{2L}|\uparrow\rangle_2 + |N\rangle_{1R}|\downarrow\rangle_1|N\rangle_{2R}|\downarrow\rangle_2\Big).\end{aligned} \tag{143}$$

Alice now looks after the matter object on the left, and Bob the one on the right. Alice immediately sends her object through the reverse Stern-Gerlach experiment, whereas Bob leaves his object alone, resulting in:

$$\begin{aligned}|\Psi_-\rangle &= |N\rangle_{1C}\Big[|\uparrow\rangle_1 \frac{1}{2}\Big(\left(|N\rangle_{2L}|\uparrow\rangle_2 + |N\rangle_{2R}|\downarrow\rangle_2\right)\Big) + |\downarrow\rangle_1 \frac{1}{2}\Big(-|N\rangle_{2L}|\uparrow\rangle_2 + |N\rangle_{2R}|\downarrow\rangle_2\Big)\Big] \\ &\equiv |N\rangle_{1C}\frac{1}{\sqrt{2}}\Big[|\leftarrow\rangle_1|N\rangle_{2L}|\uparrow\rangle_2 + |\rightarrow\rangle_1|N\rangle_{2R}|\downarrow\rangle_2\Big],\end{aligned}$$

where $|\uparrow\rangle, |\downarrow\rangle = \frac{1}{\sqrt{2}}(|\rightarrow\rangle \pm |\leftarrow\rangle)$. Before the experiment, Alice tells Bob that as soon as her object goes through the reverse Stern-Gerlach, she will perform a spin measurement in either the $\{\uparrow, \downarrow\}$ basis or the $\{\rightarrow, \leftarrow\}$ basis. She will then immediately communicate to Bob which basis she decided on. Bob then performs a position measurement on his object at a time t_B just before the signal from Alice arrives.

If Alice performs her measurement in the $\{\uparrow, \downarrow\}$ basis, then Bob's object is in a superposition of left and right and, in the case that gravity is simply sourced by the quantum expectation of matter (and within the full non-perturbative, non-linear regime), these states of matter will be 'attracted' towards each other through a classical gravitational force, as if there were physically two matter objects at the left and right positions - see $\Phi_{C2}(\boldsymbol{x})$ in (52). This then results in a change to the positions of the two states. If, on the other hand, Alice performs her measurement in the $\{\rightarrow, \leftarrow\}$ basis, then Bob's matter object will no longer be in a superposition and the gravitational potential is just that of an object at either the left or right position (depending on Alice's measurement result), such that there is no movement of the object. Therefore, by measuring the position of his matter object and checking if the object has shifted in position, Bob is, in principle, able to detect which measurement basis Alice chose, and before he receives Alice's signal. Furthermore, although we are working in the non-relativistic limit of gravity here, this would still apply if we had taken a relativistic stance since wavefunction collapse is 'instantaneous' in 'standard' quantum mechanics.

Since the gravitational force between the states is small, we can approximate the distance which Bob's object moves by $\delta x = GMt_B^2/(\Delta x)^2$, where we have assumed that the time at which Alice performs her measurement is not long after the state (142) has been created. If t_B is just before the signal from Alice reaches Bob, we can take $t_B \approx d/c$, where d is the distance between the centre of

the two masses 1 and 2, resulting in $\delta x \approx GMd^2/(c\Delta x)^2$. Taking the experimental values of Ref. [5], where $d = 450\,\mu$m, $\Delta x = 250\,\mu$m and $M = 10^{-14}$ kg, we find $\delta x \approx 10^{-41}$ m. This is smaller than the Planck length and clearly beyond current or foreseeable technological abilities. It may also be theoretically impossible to observe [58], pointing to the idea that there may be a principle that forbids using classical gravity to perform superluminal signalling [51]. Here, we assumed that the Newtonian potential is being sourced by the expectation of the quantum matter states. If instead, we chose them to be sourced by the expectation of the 'local matter states' [20] then we are effectively making the measurement process for gravity a relativistic process [22]. That is, when Alice performs her measurement, this no longer immediately updates the gravitational potentials $\Phi_{\kappa i}(\boldsymbol{x})$ because they are now defined as coming from the expectation of the matter states that are only updated by measurement processes in the past light cone of the matter objects:

$$\Phi_\kappa(\boldsymbol{x}) = -\frac{G}{c^2}\int d^3\boldsymbol{y}\frac{\mathrm{Tr}\left(\rho_{loc}^{(\kappa)}\,\hat{T}_{00}(\boldsymbol{y})\right)}{|\boldsymbol{x}-\boldsymbol{y}|}, \tag{144}$$

where $\rho_{loc}^{(\kappa)}$ is defined by taking the joint state of the system but allowing for only measurements in the past light cone of object κ and then tracing out the other object [20, 21]. In this case, irrespective of the measurement basis Alice uses, the effective gravitational potential of Bob's object remains the same up until time d/c, and so it feels a force and is displaced from its original position independent of Alice's measurement basis (up until time d/c). Therefore, even in principle, Alice and Bob are not able to perform superluminal signalling. As argued above, using a relativistic wavefunction collapse prescription in the gravity sector does not change the results of Section 2 since no measurement is performed before the matter states are brought back together in the reverse Stern-Gerlach, as in Ref. [5]. Measurements are only carried out at the end of the experiment to determine if the spins of the objects are entangled. In considering local matter states, we have assumed that it is only in the gravity sector that the collapse process is relativistic (it only changes how we update the gravitational potentials), and so this does not affect the final measurements on the spins and the deduction of whether the objects are entangled. It could be possible to extend this so that the collapse process is always relativistic such that we have causal quantum theory [20, 59]. In this case, the final measurements would have to be performed outside of each others' light cones to test for entanglement, since collapse is now always a relativistic process and not just in the gravity sector. There are questions over whether casual quantum theory, where normal quantum mechanics is updated with a relativistic collapse process, is consistent with current experiments [59]. In contrast, no experiment has tested relativistic collapse in just the gravity sector [20–22]. Yet another option to avoid superluminal signalling, at least in the above example, would be to define the potentials of each object in terms of just the standard reduced states of the respective objects rather than the full state vector:

$$\Phi_\kappa(\boldsymbol{x}) = -\frac{G}{c^2}\int d^3\boldsymbol{y}\frac{\mathrm{Tr}\left(\rho_\kappa\,\hat{T}_{00}(\boldsymbol{y})\right)}{|\boldsymbol{x}-\boldsymbol{y}|}, \tag{145}$$

where $\rho_\kappa := \mathrm{Tr}_{\lambda\neq\kappa}(\rho)$ with $\kappa, \lambda \in 1, 2$. Then there is no need to introduce a 'relativistic' version of collapse.

The stochastic theories of classical gravity considered in Section 4 are linear theories and so the arguments for superluminal signalling processes occurring in non-linear theories do not apply. These theories, however, tend to come with violations of energy conservation, which is not considered a theoretical inconsistency unless there is experimental evidence to rule this out [60, 61]. Interestingly, the consistency of these theories in explaining simple classical observations, such as spacecrafts undergoing slingshot manoeuvrers, as also been questioned recently by considering scattering processes in these theories [62]. Furthermore, to avoid infinite divergences, the decoherence processes involved in the theories also need to effectively 'smear' matter [38, 43] (there is continuous monitoring of a smeared mass density), which, together with quantum correlations of fictitious detectors, can lead to non-local processes [38, 39, 43]. However, as discussed in Section 4, when discounting these non-local effects, the virtual matter process considered here still results in entanglement. There is also a debate on

the full-consistency of a relativistic stochastic theory due to potential open problems associated with reconciling Markovian decoherence and diffusion with relativity [63–65].

We should also contrast open problems with classical theories of gravity with those of quantum gravity. The historical approach to quantizing the other interactions, electromagnetism and the weak and strong interactions (perturbative QFT), fails with gravity, leading to a non-renormalizable theory. This full theory is thus *self-inconsistent*. However, at low energy scales we can use the prescription of effective QFT, and all realistic theories of quantum gravity are thought to approximate perturbative quantum gravity in such a regime. This is was used in Section 1 since the experiment is very much working within the low-energy regime. There is a connection here with classical gravity: although it has been argued that certain fundamental classical gravity involve potential self-inconsistencies, such as superluminal signalling, we have seen how these effects are beyond the regime of the experiment and do not affect physical predictions in the regime of the experiment. Currently, there are many ideas and theories for a fully self-consistent quantum gravity theory, with no general consensus on what the true quantum gravity theory should be, if indeed gravity is quantized. The two most prominent theories are String Theory and Loop Quantum Gravity. As with classical gravity theories, the correctness of these theories as theories of nature, have also been questioned. For example, String Theory has received questions on the difficulty of formulating a fully background-independent version, and the prediction of extra dimensions [66–69]. Similarly, question marks over Loop Quantum Gravity include a lack of clear emergence of classical general relativity, and the uniqueness of the theory [70].

5.1 'Local' semi-classical gravity

As discussed above, there is a debate on whether traditional semi-classical gravity is inconsistent due to the possibility of introducing superluminal signalling processes. This is known to occur in non-linear modifications of quantum mechanics, which traditional semi-classical gravity is an example of. However, since our results are within the linear regime of the theory, as discussed in the previous section, such processes do not contribute to the entanglement effect we are considering in Section 2, which would question the locality of the process. It was also shown that such superluminal signalling processes are far beyond the regime of future experiments (and in fact may not be possible in any physical experiment [51]). However, as we saw above, it is also possible to avoid superluminal signalling processes outright by small modifications to traditional semi-classical gravity. Here, we discuss in more detail how the results of Section 2 are unchanged if we modified traditional semi-classical gravity to avoid superluminal signalling by (i) making the collapse postulate relativistic [20, 22], (ii) relativistic in just the gravity sector [22], (iii) sourcing gravity from 'local' states of matter [20, 21], or (iv) souring gravity from the reduced density matrices of matter.

It is clear that the results are unmodified by options (i) and (ii) since no measurement process occurs for the period of time where we analyse whether entanglement is created in Section 2. Measurements only occur at the end of (many runs of) the experiment to determine whether there is entanglement between the objects. In the case of (i), this would mean that the experimentalist must make sure to perform the measurements on the objects inside each other's light cones [59] such that there is sufficient time for the measurement result of one object to 'propagate' to the other. This is not an issue for future experiments since this level of control will be outside current technology [9]. For option (ii), no such requirement on the final measurements is needed since at the end of the experiment the entanglement is transferred solely to the spin sector and the masses are not in position superposition states.

We now first discuss option (iv) before (iii). In traditional semi-classical gravity, the Newtonian potential is defined by (52), where $\hat{T}_{00} = \hat{\pi}\hat{\pi}^\dagger - \partial_i\hat{\phi}\partial^i\hat{\phi} + m^2c^2\hat{\phi}^\dagger\hat{\phi}/\hbar^2$. As described in Section 2.2, we only need consider the initial state $|\psi\rangle$ in calculating $\Phi(\boldsymbol{x})$, which is given by Equation 5 in the main text ((142) above). That is, using the orthonormality of the states, we have:

$$\langle\hat{T}_{00}\rangle = \frac{1}{4}\sum_{i,j} {}_{1i}\langle N|\,{}_{2j}\langle N|\hat{T}_{00}|N\rangle_{1i}|N\rangle_{2j}. \tag{146}$$

Then, given the non-relativistic approximation $R \gg \hbar/(mc)$, we can use (23) (without the time dependence) and discount the spatial derivatives in $\hat{T}_{00}$. The first and third terms in $\hat{T}_{00}$ then contribute the same and we end up with:

$$\langle \hat{T}_{00} \rangle = \frac{1}{2} \frac{2m^2c^2}{\hbar^2} \sum_{\kappa,i} {}_{\kappa i}\langle N|\hat{\phi}^\dagger \hat{\phi}|N\rangle_{\kappa i}. \tag{147}$$

Plugging in the contractions (23), we then end up with (52):

$$\Phi(\boldsymbol{x}) = \Phi_{C1}(\boldsymbol{x}) + \Phi_{C2}(\boldsymbol{x}), \tag{148}$$

where

$$\Phi_{C\kappa}(\boldsymbol{x}) := \frac{1}{2} \left(\Phi_{\kappa L}(\boldsymbol{x}) + \Phi_{\kappa R}(\boldsymbol{x}) \right), \tag{149}$$

which is the result expected for traditional semi-classical gravity - the potential for each matter object is the average of its potentials for the two superposition states.

In option (iv), we replace the definition of $\Phi(\boldsymbol{x})$ with $\Phi(\boldsymbol{x}) = \Phi_1(\boldsymbol{x}) + \Phi_2(\boldsymbol{x})$ where $\Phi_\kappa(\boldsymbol{x})$ is given by (145) above. Using (23) again and $R \gg \hbar/(mc)$, we end up with the same expression for $\Phi(\boldsymbol{x})$ as in traditional semi-classical gravity. This is easy to see from the fact that the initial state is a product state: $|\psi\rangle = |\psi\rangle_1 \otimes |\psi\rangle_2$ and so $\mathrm{Tr}(\rho_1 \hat{T}_{00}) + \mathrm{Tr}(\rho_2 \hat{T}_{00}) = {}_1\langle\psi|\hat{T}_{00}|\psi\rangle_1 + {}_2\langle\psi|\hat{T}_{00}|\psi\rangle_2$. Here, $|\psi\rangle_\kappa$ is the state vector for object κ, i.e. $|\psi\rangle_\kappa = \frac{1}{\sqrt{2}}(|N\rangle_{\kappa L} + |N\rangle_{\kappa R})$. Then, from the orthonormality of the states, we clearly end up with (147) above and thus (52). In general, in fact, since ${}_{\kappa i}\langle N|\hat{T}_{00}|N\rangle_{\kappa j} = 0$ for $i \neq j$, we should expect the only difference between the definitions of $\Phi(\boldsymbol{x})$ for this 'local' case and traditional semi-classical gravity to occur when there is a measurement process, and as described above, this only occurs right at the end of the experiment and so does not affect the entanglement calculation in Section 2. Option (iii) is to change the definition of $\Phi_\kappa(\boldsymbol{x})$ to be (144). The only difference is to trace out all measurement outcomes outside of the object's light cone. Therefore, since there is no measurement until the end of the experiment, again, nothing changes for the calculation in Section 2.

It is not surprising that the entanglement effect is the same in traditional semi-classical gravity as with the above modifications since the entanglement process is not associated with a superluminal signalling effect, and is in fact a local process. Furthermore, the entanglement is not coming from the form of $\Phi(\boldsymbol{x})$, which cannot be in a superposition in classical gravity, and instead from the fact that there is a superposition of virtual matter propagators from the classical gravity interaction.

6 Alternative signatures of quantum gravity to entanglement

That there are ways for entanglement to be generated from non-quantum and local theories of gravity raises the question of whether there are any signatures that can *only* ever be generated by quantum gravity theories. Alternative signatures to entanglement for evidencing quantum gravity include a measurement inequality [71], but since this inequality derives from considering classical gravity as LOCC, the virtual matter process considered here would also be expected to violate the inequality in general. Another alternative that has been considered is non-Gaussianity or Wigner negativity [72]. This is thought not to be based on LOCC and instead relies on the idea that, when the gravitational field has no associated quantum operator, the theory preserves Gaussianity since the Hamiltonian is quadratic in quantum field matter operators. Since the entangling virtual matter process considered here derives from (39), with $\hat{H}_{int}$ defined by Equation 4 in the main text, it is Gaussian and so non-Gaussianity would be expected to be a signal of only quantum gravity in this context. However, the work here demonstrates that we do not need general proofs that certain signatures can only ever be associated with quantum gravity, we just need *strong evidence*, as discussed in the main text.

References

[1] Peskin, M.E., Schroeder, D.V.: An Introduction to Quantum Field Theory. Addison-Wesley, Reading, USA (1995). https://doi.org/10.1201/9780429503559

[2] Gupta, S.N.: Quantization of Einstein's Gravitational Field: Linear Approximation. Proceedings of the Physical Society. Section A **65**(3), 161 (1952) https://doi.org/10.1088/0370-1298/65/3/301

[3] Gupta, S.N.: Supplementary conditions in the quantized gravitational theory. Phys. Rev. **172**, 1303–1307 (1968) https://doi.org/10.1103/PhysRev.172.1303

[4] Pavšič, M.: Localized states in quantum field theory. Advances in Applied Clifford Algebras **28**(5), 1–29 (2018) https://doi.org/10.1007/s00006-018-0904-5

[5] Bose, S., Mazumdar, A., Morley, G.W., Ulbricht, H., Toroš, M., Paternostro, M., Geraci, A.A., Barker, P.F., Kim, M.S., Milburn, G.: Spin Entanglement Witness for Quantum Gravity. Phys. Rev. Lett. **119**, 240401 (2017) https://doi.org/10.1103/PhysRevLett.119.240401

[6] Marletto, C., Vedral, V.: Gravitationally Induced Entanglement between Two Massive Particles is Sufficient Evidence of Quantum Effects in Gravity. Phys. Rev. Lett. **119**, 240402 (2017) https://doi.org/10.1103/PhysRevLett.119.240402

[7] Maggiore, M.: Gravitational Waves Volume 1: Theory and Experiments. Oxford University Press, Oxford (2008)

[8] DeWitt, B.S.: Quantum Theory of Gravity. II. The Manifestly Covariant Theory. Phys. Rev. **162**, 1195–1239 (1967) https://doi.org/10.1103/PhysRev.162.1195

[9] Christodoulou, M., Di Biagio, A., Aspelmeyer, M., Brukner, Č., Rovelli, C., Howl, R.: Locally mediated entanglement in linearized quantum gravity. Phys. Rev. Lett. **130**, 100202 (2023) https://doi.org/10.1103/PhysRevLett.130.100202

[10] Mari, A., De Palma, G., Giovannetti, V.: Experiments Testing Macroscopic Quantum Superpositions Must Be Slow. Scientific Reports **6**(1), 22777 (2016) https://doi.org/10.1038/srep22777

[11] Aspelmeyer, M.: In: Kiefer, C. (ed.) When Zeh meets Feynman: How to avoid the appearance of a classical World in gravity experiments, pp. 85–95. Springer, Cham (2022). https://doi.org/10.1007/978-3-030-88781-0_5

[12] Vidal, G., Werner, R.F.: Computable measure of entanglement. Phys. Rev. A **65**, 032314 (2002) https://doi.org/10.1103/PhysRevA.65.032314

[13] Anastopoulos, C., Hu, B.-L.: Comment on "A Spin Entanglement Witness for Quantum Gravity" and on "Gravitationally Induced Entanglement Between Two Massive Particles Is Sufficient Evidence of Quantum Effects in Gravity". https://doi.org/10.48550/arXiv.1804.11315

[14] Anastopoulos, C., Lagouvardos, M., Savvidou, K.: Gravitational effects in macroscopic quantum systems: a first-principles analysis. Classical and Quantum Gravity **38**(15), 155012 (2021) https://doi.org/10.1088/1361-6382/ac0bf9

[15] Martín-Martínez, E., Perche, T.R.: What gravity mediated entanglement can really tell us about quantum gravity. Phys. Rev. D **108**, 101702 (2023) https://doi.org/10.1103/PhysRevD.108.L101702

[16] Fragkos, V., Kopp, M., Pikovski, I.: On inference of quantization from gravitationally induced entanglement. AVS Quantum Science **4**(4), 045601 (2022) https://doi.org/10.1116/5.0101334

[17] Møller, C.: Les théories relativistes de la gravitation. Colloques Internationaux CNRS **91**(1), 15–29 (1962)

[18] Rosenfeld, L.: On quantization of fields. Nuclear Physics **40**, 353–356 (1963) https://doi.org/10.1016/0029-5582(63)90279-7

[19] Carney, D., Stamp, P.C.E., Taylor, J.M.: Tabletop experiments for quantum gravity: a user's manual. Classical and Quantum Gravity **36**(3), 034001 (2019) https://doi.org/10.1088/1361-6382/aaf9ca

[20] Kent, A.: Nonlinearity without superluminality. Physical Review A **72**(1), 012108 (2005) https://doi.org/10.1103/PhysRevA.72.012108

[21] Kent, A.: Simple refutation of the Eppley–Hannah argument. Classical and Quantum Gravity **35**(24), 245008 (2018) https://doi.org/10.1088/1361-6382/aaea20

[22] Helou, B., Chen, Y.: Extensions of Born's rule to non-linear quantum mechanics, some of which do not imply superluminal communication. Journal of Physics: Conference Series **880**(1), 012021 (2017) https://doi.org/10.1088/1742-6596/880/1/012021

[23] Christodoulou, M., Biagio, A.D., Howl, R., Rovelli, C.: Gravity entanglement, quantum reference systems, degrees of freedom. Classical and Quantum Gravity **40**(4), 047001 (2023) https://doi.org/10.1088/1361-6382/acb0aa

[24] Bose, S., Mazumdar, A., Schut, M., Toroš, M.: Mechanism for the quantum natured gravitons to entangle masses. Phys. Rev. D **105**, 106028 (2022) https://doi.org/10.1103/PhysRevD.105.106028

[25] Howl, R., Penrose, R., Fuentes, I.: Exploring the unification of quantum theory and general relativity with a Bose–Einstein condensate. New J. Phys. **21**(4), 043047 (2019) https://doi.org/10.1088/1367-2630/ab104a

[26] Marletto, C., Vedral, V.: Witnessing nonclassicality beyond quantum theory. Phys. Rev. D **102**, 086012 (2020) https://doi.org/10.1103/PhysRevD.102.086012

[27] Galley, T.D., Giacomini, F., Selby, J.H.: A no-go theorem on the nature of the gravitational field beyond quantum theory. Quantum **6**, 779 (2022) https://doi.org/10.22331/q-2022-08-17-779

[28] Krisnanda, T., Zuppardo, M., Paternostro, M., Paterek, T.: Revealing Non-Classicality of Inaccessible Objects. Phys. Rev. Lett. **119**(12), 120402 (2017) https://doi.org/10.1103/physrevlett.119.120402 arXiv:1607.01140

[29] Andersen, T.C.: Quantum statistics in Bohmian trajectory gravity. Journal of Physics: Conference Series **1275**(1), 012038 (2019) https://doi.org/10.1088/1742-6596/1275/1/012038

[30] Döner, M.K., Großardt, A.: Is gravitational entanglement evidence for the quantization of spacetime? Foundations of Physics **52**(5), 101 (2022) https://doi.org/10.1007/s10701-022-00619-0

[31] Kent, A.: Should We Necessarily Treat Masses as Localized When Analysing Tests of Quantum Gravity? https://doi.org/10.48550/arXiv.2405.20514

[32] Franzmann, G.: To Be or Not to Be, but Where? https://doi.org/10.48550/arXiv.2405.21031

[33] Marchese, M.M., Plávala, M., Kleinmann, M., Nimmrichter, S.: Newton's laws of motion generating gravity-mediated entanglement. Phys. Rev. A **111**, 042202 (2025) https://doi.org/10.1103/PhysRevA.111.042202

[34] Feynman, R.: The role of gravitation in physics. In: DeWitt, C.M., Rickles, D. (eds.) Chapel Hill Conference Proceedings, pp. 250–256. Edition Open Access, North Carolina (1957). https://doi.org/10.34663/9783945561294-00 . http://www.edition-open-sources.org/sources/5/index.html

[35] Kafri, D., Taylor, J.: A noise inequality for classical forces. arXiv preprint arXiv:1311.4558 (2013) https://doi.org/https://arxiv.org/abs/1311.4558 [quant-ph]

[36] Huggett, N., Linnemann, N., Schneider, M.D.: Quantum Gravity in a Laboratory? Elements in the Foundations of Contemporary Physics. Cambridge University Press, Cambridge (2023). https://doi.org/10.1017/9781009327541

[37] Telali, E., Perche, T.R., Martín-Martínez, E.: Causality in relativistic quantum interactions without mediators. Phys. Rev. D **111**, 085005 (2025) https://doi.org/10.1103/PhysRevD.111.085005

[38] Tilloy, A., Diósi, L.: Sourcing semiclassical gravity from spontaneously localized quantum matter. Phys. Rev. D **93**, 024026 (2016) https://doi.org/10.1103/PhysRevD.93.024026

[39] Trillo, D., Navascués, M.: Diósi-Penrose model of classical gravity predicts gravitationally induced entanglement. Phys. Rev. D **111**, 121101 (2025) https://doi.org/10.1103/PhysRevD.111.L121101

[40] Page, D.N., Geilker, C.D.: Indirect Evidence for Quantum Gravity. Phys. Rev. Lett. **47**, 979–982 (1981) https://doi.org/10.1103/PhysRevLett.47.979

[41] Diósi, L.: A universal master equation for the gravitational violation of quantum mechanics. Physics Letters A **120**(8), 377–381 (1987) https://doi.org/10.1016/0375-9601(87)90681-5

[42] Oppenheim, J.: A Postquantum Theory of Classical Gravity? Phys. Rev. X **13**, 041040 (2023) https://doi.org/10.1103/PhysRevX.13.041040

[43] Layton, I., Oppenheim, J., Russo, A., Weller-Davies, Z.: The weak field limit of quantum matter back-reacting on classical spacetime. Journal of High Energy Physics **2023**(8), 1–43 (2023) https://doi.org/10.1038/s41467-023-43348-2

[44] Bassi, A., Großardt, A., Ulbricht, H.: Gravitational decoherence. Classical and Quantum Gravity **34**(19), 193002 (2017) https://doi.org/10.1088/1361-6382/aa864f

[45] Tilloy, A., Diósi, L.: Principle of least decoherence for Newtonian semiclassical gravity. Phys. Rev. D **96**, 104045 (2017) https://doi.org/10.1103/PhysRevD.96.104045

[46] Diósi, L.: Models for Universal Reduction of Macroscopic Quantum Fluctuations. Phys. Rev. A **40**(3), 1165–1174 (1989) https://doi.org/10/fv2d9m

[47] Penrose, R.: On Gravity's Role in Quantum State Reduction. General Relativity and Gravitation **28**, 581–600 (1996) https://doi.org/10/d52jm5

[48] Eppley, K., Hannah, E.: The necessity of quantizing the gravitational field. Foundations of Physics **7**(1), 51–68 (1977) https://doi.org/10.1007/BF00715241

[49] Gisin, N.: Weinberg's non-linear quantum mechanics and supraluminal communications. Physics Letters A **143**(1), 1–2 (1990) https://doi.org/10.1016/0375-9601(90)90786-N

[50] Huggett, N., Callender, C.: Why quantize gravity (or any other field for that matter)? Philosophy of Science **68**(S3), 382–394 (2001) https://doi.org/10.1086/392923

[51] Mattingly, J.: Why Eppley and Hannah's thought experiment fails. Physical Review D—Particles, Fields, Gravitation, and Cosmology **73**(6), 064025 (2006) https://doi.org/10.1103/PhysRevD.73.

064025

[52] Ballentine, L.E.: The Statistical Interpretation of Quantum Mechanics. Rev. Mod. Phys. **42**, 358–381 (1970) https://doi.org/10.1103/RevModPhys.42.358

[53] Rozema, L.A., Darabi, A., Mahler, D.H., Hayat, A., Soudagar, Y., Steinberg, A.M.: Violation of heisenberg's measurement-disturbance relationship by weak measurements. Phys. Rev. Lett. **109**, 100404 (2012) https://doi.org/10.1103/PhysRevLett.109.100404

[54] Bahrami, M., Großardt, A., Donadi, S., Bassi, A.: The Schrödinger–Newton equation and its foundations. New Journal of Physics **16**(11), 115007 (2014) https://doi.org/10.1088/1367-2630/16/11/115007

[55] Kent, A.: Causality in time-neutral cosmologies. Physical Review D **59**(4), 043505 (1998) https://doi.org/10.48550/arXiv.gr-qc/9703041

[56] Giulini, D., Großardt, A., Schwartz, P.K.: In: Pfeifer, C., Lämmerzahl, C. (eds.) Coupling Quantum Matter and Gravity, pp. 491–550. Springer, Cham (2023). https://doi.org/10.1007/978-3-031-31520-6_16

[57] Mielnik, B.: Comments on: "Weinberg's Nonlinear Quantum Mechanics and Einstein-Podolsky-Rosen paradox", by Joseph Polchinski. arXiv preprint quant-ph/0012041 (2000) https://doi.org/10.48550/arXiv.quant-ph/0012041

[58] Mead, C.A.: Possible connection between gravitation and fundamental length. Physical Review **135**(3B), 849 (1964) https://doi.org/10.1103/PhysRev.135.B849

[59] Kent, A.: Testing causal quantum theory. Proceedings of the Royal Society A **474**(2220), 20180501 (2018) https://doi.org/10.1098/rspa.2018.0501

[60] Donadi, S., Piscicchia, K., Curceanu, C., Diósi, L., Laubenstein, M., Bassi, A.: Underground test of gravity-related wave function collapse. Nature Physics **17**(1), 74–78 (2021) https://doi.org/10.1038/s41567-020-1008-4

[61] Oppenheim, J., Sparaciari, C., Šoda, B., Weller-Davies, Z.: Gravitationally induced decoherence vs space-time diffusion: testing the quantum nature of gravity. Nature Communications **14**(1), 7910 (2023) https://doi.org/10.1038/s41467-023-43348-2

[62] Carney, D., Matsumura, A.: Classical-quantum scattering. Classical and Quantum Gravity **42**(13), 135010 (2025) https://doi.org/10.1088/1361-6382/ade589

[63] Diósi, L.: Classical-quantum hybrid canonical dynamics and its difficulties with special and general relativity. Physical Review D **110**(8), 084052 (2024)

[64] Diósi, L.: Is there a relativistic Gorini-Kossakowski-Lindblad-Sudarshan master equation? Phys. Rev. D **106**, 051901 (2022) https://doi.org/10.1103/PhysRevD.106.L051901

[65] Grudka, A., Morris, T.R., Oppenheim, J., Russo, A., Sajjad, M.: Renormalisation of postquantum-classical gravity. arXiv:2402.17844 (2024) https://doi.org/10.48550/arXiv.2402.17844

[66] Penrose, R.: Fashion, Faith, and Fantasy in the New Physics of the Universe. Princeton University Press, Princeton, NJ, USA (2016). https://doi.org/10.1515/9781400880287 . http://www.jstor.org/stable/j.ctvc775bn

[67] Smolin, L.: The Trouble with Physics: The Rise of String Theory, the Fall of a Science, and What Comes Next. Houghton Mifflin Harcourt, Boston, MA (2007)

[68] Woit, P.: Not Even Wrong: The Failure of String Theory and the Search for Unity in Physical Law. Basic Books, New York, NY, USA (2006). https://www.basicbooks.com/titles/peter-woit/not-even-wrong/9780465092765/

[69] Schwarz, J.H.: The status of string theory. arXiv preprint hep-th/9711029 (1997) https://doi.org/10.48550/arXiv.hep-th/9711029

[70] Nicolai, H., Peeters, K., Zamaklar, M.: Loop quantum gravity: an outside view. Classical and Quantum Gravity **22**(19), 193 (2005) https://doi.org/10.1088/0264-9381/22/19/R01

[71] Lami, L., Pedernales, J.S., Plenio, M.B.: Testing the quantumness of gravity without entanglement. Phys. Rev. X **14**, 021022 (2024) https://doi.org/10.1103/PhysRevX.14.021022

[72] Howl, R., Vedral, V., Naik, D., Christodoulou, M., Rovelli, C., Iyer, A.: Non-Gaussianity as a signature of a quantum theory of gravity. PRX Quantum **2**(1), 010325 (2021) https://doi.org/10/gkq6wg arXiv:2004.01189
